# Extreme value methods for estimating rare events in Utopia: EVA (2023) conference data challenge: team Lancopula Utopiversity

**DOI:** 10.1007/s10687-024-00498-w

**Published:** 2024-11-22

**Authors:** Lídia Maria André, Ryan Campbell, Eleanor D’Arcy, Aiden Farrell, Dáire Healy, Lydia Kakampakou, Conor Murphy, Callum John Rowlandson Murphy-Barltrop, Matthew Speers

**Affiliations:** 1 STOR-i Centre for Doctoral Training, Lancaster University, Lancaster, LA1 4YR United Kingdom; 2 School of Mathematical Sciences, Lancaster University, Lancaster, LA1 4YF United Kingdom; 3 Environment Agency, Lutra House, Dodd Way Off Seedlee Road, Walton Summit Centre, Preston, PR5 8BX United Kingdom; 4 Dipartimento di Scienze Ambientali, Informatica e Statistica, Università Ca’ Foscari Venezia, Campus Scientifico, via Torino 155, Mestre Venezia, 30172 Italia; 5 Institut Für Mathematische Stochastik, Technische Universität Dresden, Helmholtzstraße 10, 01069 Dresden, Germany; 6 Center for Scalable Data Analytics and Artificial Intelligence (ScaDS.AI), Dresden/Leipzig, Germany

**Keywords:** Extremal dependence, Generalised additive modelling, Non-stationary extremes, Peaks-over-threshold modelling, 62G32

## Abstract

To capture the extremal behaviour of complex environmental phenomena in practice, flexible techniques for modelling tail behaviour are required. In this paper, we introduce a variety of such methods, which were used by the Lancopula Utopiversity team to tackle the EVA (2023) Conference Data Challenge. This data challenge was split into four challenges, labelled C1-C4. Challenges C1 and C2 comprise univariate problems, where the goal is to estimate extreme quantiles for a non-stationary time series exhibiting several complex features. For these, we propose a flexible modelling technique, based on generalised additive models, with diagnostics indicating generally good performance for the observed data. Challenges C3 and C4 concern multivariate problems where the focus is on estimating joint probabilities. For challenge C3, we propose an extension of available models in the multivariate literature and use this framework to estimate joint probabilities in the presence of non-stationary dependence. Finally, for challenge C4, which concerns a 50-dimensional random vector, we employ a clustering technique to achieve dimension reduction and use a conditional modelling approach to estimate extremal probabilities across independent groups of variables.

**Supplementary Information:**

The online version contains supplementary material available at 10.1007/s10687-024-00498-w.

## Introduction

This paper details an approach to the data challenge organised for the Extreme Value Analysis (EVA) 2023 Conference. The objective of the challenge was to estimate extremal probabilities, or their associated quantiles, for simulated environmental data sets for various locations in a fictitious country called Utopia. The data challenge is split into 4 challenges; challenges C1 and C2 focus on a setting where data is obtained from a single location while challenges C3 and C4 concern multivariate data sets, where data is obtained simultaneously from multiple locations.

Challenge C1 requires estimation of the 0.9999-quantile of the distribution of the environmental response variable *Y* conditional on a covariate vector $\varvec{X}$, for 100 realisations of covariates. To do so, we model the tail of $Y\mid \varvec{X}=\varvec{x}$ using a generalised Pareto distribution (GPD; Pickands 1975) and employ the extreme value generalised additive modelling (EVGAM) framework, first introduced by Youngman (2019), to account for the non-stationary data structure. We consider a variety of model formulations and select our final model using cross-validation. Furthermore, central 50% confidence intervals are estimated via a non-stationary bootstrapping technique, and the final model performance is assessed using the number of times the true conditional quantile lies in the confidence intervals (Rohrbeck et al. 2023). For Challenge C2, we are interested in estimating the value of *q* that satisfies $\Pr (Y>q)={1}/{(300T)}$, where $T = 200$.

Challenges C3 and C4 concern the estimation of probabilities for extreme multivariate regions, subsets of $\mathbb {R}^d$, where some or all of the components are so large that we seldom observe any data in them. Such estimates require techniques for modelling and extrapolating within the joint tail. For challenge C3, we want to estimate two joint tail probabilities for three unknown non-stationary environmental variables. To achieve this, we propose a non-stationary extension of the model introduced by Wadsworth and Tawn (2013). Lastly, for challenge C4, we wish to estimate the probability that 50 variables (locations) jointly exceed prespecified extreme thresholds. Based on an initial analysis, we separate the variables into five independent groups, and obtain distinct probability estimates for each group using the conditional extremes approach of Heffernan and Tawn (2004).

The remainder of the paper is structured as follows. A suitable background to EVA is provided in Section 2, introducing concepts required throughout our work. Section 3 covers our approach to the univariate challenges C1 and C2, and the multivariate challenges C3 and C4 are considered in Sections 4 and 5, respectively. The paper ends with a discussion of the results of all challenges in Section 6.

## EVA background

### Univariate modelling

Univariate EVA methods are concerned with capturing the behaviour of the tail of a distribution which allows for extreme quantities to be estimated. A common univariate approach is the peaks-over-threshold framework. Consider a continuous, independent and identically distributed (IID) random variable *Y* with distribution function *F* and upper endpoint $y^F := \text {sup}\{y : F(y) < 1\}$. Pickands (1975) shows that, for some high threshold $v < y^F$, the excesses $(Y - v) \mid Y>v$, after suitable rescaling, converge in distribution to a GPD as $v \rightarrow y^F$. Davison and Smith (1990) provide an overview of the properties of the GPD, and also propose an extension of this framework to the non-stationary setting: given a non-stationary process *Y* with associated covariate(s) $\varvec{X}$, the authors propose the following model

2.1 $$\begin{aligned} \Pr (Y> y + v \mid Y > v, \varvec{X}=\varvec{x}) = \left( 1 + \frac{y\xi (\varvec{x})}{\sigma (\varvec{x})}\right) _+^{-1/\xi (\varvec{x})}, \end{aligned}$$

for $y>0$, where $\sigma (\cdot ), \xi (\cdot )$ are the covariate-dependent scale and shape parameters, respectively. Recent extensions of the Davison and Smith (1990) framework include allowing the threshold to be covariate-dependent, i.e., $v(\varvec{x})$ (Kyselý et al. 2010; Northrop and Jonathan 2011), and using generalised additive models (GAMs; Chavez-Demoulin and Davison 2005, Youngman 2019) to capture the functions $\sigma (\cdot )$ and $\xi (\cdot )$ in a flexible manner.

### Extremal dependence measures

In addition to analysing marginal tail behaviours, multivariate EVA methods are concerned with quantifying the dependence between extremes of the individual components. An important classification of this dependence is obtained through the measure $\chi$ (Joe 1997): given a *d*-dimensional random vector $\varvec{Z}$, with $d\ge 2$ and $Z_i \sim F$ for all $i \in \{1,\ldots ,d\}$,

2.2 $$\begin{aligned} \chi (u) := \left( \frac{1}{1-u}\right) \Pr ( F(Z_1)> u, \dots , F(Z_d) > u), \end{aligned}$$

with $u \in [0,1)$. Where the limit exists, we set $\chi := \lim _{u \rightarrow 1}\chi (u) \in [0,1]$. When $\chi > 0$, we say that the variables in $\varvec{Z}$ exhibit asymptotic dependence, i.e., can take their largest values simultaneously, with the strength of dependence increasing as $\chi$ approaches 1. If $\chi = 0$, the variables cannot all take their largest values together. In particular, for $d=2$, we refer to the case $\chi = 0$ as asymptotic independence.

We also consider the coefficient of tail dependence proposed by Ledford and Tawn (1996). Using the formulation given in Resnick (2002), let

$$\begin{aligned} \eta (u):=\frac{\log \left( 1-u\right) }{\log \Pr \left( F(Z_1)> u, \dots , F(Z_d) > u\right) }, \end{aligned}$$

with $u \in [0,1)$. When the limit exists, we set $\eta :=\lim _{u\rightarrow 1}\eta (u) \in (0,1]$. The cases $\eta = 1$ and $\eta < 1$, correspond to $\chi > 0$ and $\chi = 0$, respectively. For $\eta <1$, this coefficient quantifies the form of dependence for random vectors that do not take their largest values simultaneously.

Since $\chi$ and $\eta$ are limiting values, they are unknown in practice and must be approximated using numerical techniques. Therefore, when quantifying extremal dependence, we approximate $\chi$ ($\eta$) using empirical estimates of $\chi (u)$ $\big (\eta (u)\big )$ for some high threshold *u*.

## Challenges C1 and C2

Both challenges concern 70 years of daily data for the capital city of Amaurot. Each year has 12 months of 25 days and two seasons (season 1 for months 1-6, and season 2 for months 7-12). Suppose *Y* is an unknown response variable, and $\varvec{X}=(V_{1},\ldots , V_{8})$ is a vector of covariates, $(V_1, V_2, V_3, V_4)$ denoting unknown environmental variables and $(V_5,V_6,V_7,V_8)$ denoting season, wind direction (radians), wind speed (unknown scale), and atmosphere (recorded monthly), respectively.

For C1, we build a model for $Y \mid \varvec{X}$ and estimate the 0.9999-quantile, with associated 50% confidence intervals, for 100 different covariate combinations denoted $\tilde{\varvec{x}}_i$ for $i \in \{1,\ldots ,100\}$. Note $\tilde{\varvec{x}}_i$ are not covariates observed within the data set, but new observations provided by the challenge organisers.

For C2, we estimate the marginal quantile *q* such that $\Pr (Y>q)=(6\times 10)^{-4}$, which corresponds to a once in 200-year event in the IID setting; in particular, *q* is obtained subject to a predefined loss function. We first estimate the marginal distribution $F_{Y}(y)$ using Monte-Carlo techniques; see for instance, Eastoe and Tawn (2009). Since we have a large sample size, $n=21,000$, it is reasonable to assume that the observed covariate sample is representative of $\varvec{X}$. Thus, we can approximate the marginal distribution $F_Y(y)$ as follows,

3.1 $$\begin{aligned} \hat{F}_{Y}(y)=\int _{\varvec{X}} F_{Y\mid {\varvec{X}}}(y\mid \varvec{x})f_{\varvec{X}}(\varvec{x})\textrm{d}\varvec{x}\approx \frac{1}{n}\sum \limits _{t=1}^{n} F_{Y_t\mid {\varvec{X}_t}}(y_t\mid \varvec{x}_t). \end{aligned}$$

where $F_{Y\mid {\varvec{X}}}(\cdot )$ is the conditional distribution function of $Y\mid \varvec{X}$ and $f_{\varvec{X}}(\cdot )$ denotes the joint probability density of the covariates $\varvec{X}$.

We incorporate the following loss function provided by the challenge organisers,

3.2 $$\begin{aligned} \mathscr {L}(q,\hat{q}) = {\left\{ \begin{array}{ll} 0.9(0.99q - \hat{q})& \text {if }\, 0.99q>\hat{q},\\ 0 & \text {if }\, \left| q-\hat{q}\right| \le 0.01q,\\ 0.1(\hat{q}-1.01q)& \text {if }\, 1.01q<\hat{q}, \end{array}\right. } \end{aligned}$$

where *q* and $\hat{q}$ are the true and estimated marginal quantiles, respectively. This loss function penalises under-estimation more heavily than an over-estimation.

We conduct the same exploratory data analysis for both challenges given the same covariates are used; this is outlined in Section 3.1. In Section 3.2 we introduce our techniques for modelling $Y \mid \varvec{X}$, which is then used for modelling *Y* via (3.1). Our approach for uncertainty quantification is outlined in Section 3.3, and we give our results for both challenges in Section 3.4.

### Exploratory data analysis

Given the covariate vector $\varvec{X_t}=\{V_{1,t},\ldots ,V_{8,t}\}$, the environmental response variable $Y_{t},\; t \in \{1,\ldots ,n\},$ is temporally independent (Rohrbeck et al. 2023). However, it is not clear which covariates affect *Y*, and what form these covariate-response relationships take. In what follows, we aim to explore these relationships so we can account for them in our modelling framework.

**Fig. 1:**
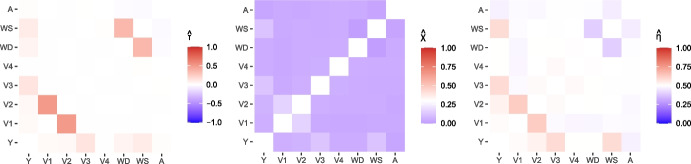
Heat maps for dependence measures for each pair of variables: Kendall’s $\tau$ (left), $\chi$ (middle) and $\eta$ (right). Note the scale in each plot varies, depending on the support of the measure, and the diagonals are left blank, where each variable is compared against itself

To begin, we explore the dependence between all variables to understand the relationships between covariates, as well as the relationships between individual covariates and the response variable. We investigate dependence in the main body of the data using Kendall’s $\tau$ measure, while for the joint tails, we use the pairwise extremal dependence coefficients $\chi$ and $\eta$ defined in Section 2; values for all pairs are shown in Fig. 1, with the threshold *u* set at the empirical 0.95-quantile for the extremal measures.

The response variable *Y* has the strongest dependence with $V_3$ in the body of the distribution (see $\hat{\tau }$ in Fig. 1), followed by $V_6$ (wind speed) then $V_7$ (wind direction). For the tail of the distribution, *Y* has strongest dependence with $V_2$, $V_3$ and $V_6$ (see $\hat{\chi }$ and $\hat{\eta }$ in Fig. 1). We also find strong dependence between $V_6$ and $V_7$ in the body, but evidence of weak dependence in the tail (dark blue for $\hat{\chi }$ and $\hat{\eta }$). There is also strong dependence between $V_1$ and $V_2$ in both the body and tail (see dark red for $\hat{\eta }$). We find very similar dependence relationships when the data are split into seasons. In the Supplementary Material, we show scatter plots of each covariate against the response variable; these demonstrate a highly non-linear relationship for each explanatory variable with *Y*.

Next, we explore temporal relationships for the response variable *Y*. We first find temporal non-stationarity as the distribution of *Y* varies significantly with $V_5$ (season); see the Supplementary Material for more detail. The mean and range of *Y* is higher in season 1 than season 2, with greater extreme values observed in season 1. However, within each season, across months, there is little temporal variation in the distribution of *Y*. We also find that *Y* exhibits temporal independence at all lags, with auto-correlation function (acf) values close to zero; see the Supplementary Material.

As noted in Rohrbeck et al. (2023), 11.7% of the observations have at least one value missing completely at random (MCAR). A detailed breakdown of the pattern of missing predictor observations is provided in the Supplementary Material. Since we can assume the data are MCAR, ignoring the observations that have a missing predictor covariate will not bias our inference, however, a complete case analysis is undesirable due to the amount of data loss. To mitigate against this, we attempted to impute the observations where predictors are missing but ultimately could not find an imputation method that satisfactorily retained the dependence structure between the response and covariates, particularly in the tails of the distribution. Therefore, we use a case analysis approach, whereby an observation is only removed if a predictor covariate of interest is missing. This results in only 4% of observations being removed for our final model.

### Methods

Due to the complex nature of the data, we consider various non-stationary GPD models, as in Eq. 2.1, that are formulated as GAMs to fit $Y\mid \varvec{X}$. For threshold selection, we extend the method proposed by Murphy et al. (2024) to select a threshold for non-stationary, covariate-dependent GPD models; the details are provided in Section 3.2.1. Our inference and model selection procedures are then provided in Sections 3.2.2 and 3.2.3, respectively. We note that the same model formulation is used for both C1 and C2 with a small adjustment to the parameter estimation procedure for C2 to incorporate the provided loss function given in Eq. 3.2. We utilise (3.1) to obtain the marginal distribution of *Y*.

#### General model formulation

Let $\tilde{\varvec{X}_t}$ denote the set of predictor covariates with $t \in \{1,\ldots ,n\}$. Then $y_{t}$ and $\tilde{\varvec{x}}_t$ denote the observations of the response variable and predictive covariates, respectively. We consider models with the following form,

3.3 $$\begin{aligned} F_{Y_t|\tilde{\varvec{X}}_t}(y_{t} | \tilde{\varvec{X}}_t = \tilde{\varvec{x}}_t) = 1-\zeta (\tilde{\varvec{x}}_t)\left[ 1+\xi (\tilde{\varvec{x}}_t)\left( \frac{y_{t}-v(\tilde{\varvec{x}}_t)}{\sigma (\tilde{\varvec{x}}_t)}\right) \right] _+^{-1/\xi (\tilde{\varvec{x}}_t)}, \end{aligned}$$

where $v(\tilde{\varvec{x}}_t)$ and $\zeta (\tilde{\varvec{x}}_t)$ are a covariate-dependent threshold and rate parameter, respectively, such that the rate parameter corresponds to the probability of exceeding the threshold.

Our analysis in Section 3.1 indicates that $V_{3}$, $V_{5}$ (season), and $V_{6}$ (wind speed) exhibit non-trivial dependence relationships with the response variable. Therefore we assume these variables can be used as predictor variables for modelling *Y*, and set $\tilde{\varvec{x}} := (\varvec{V}_{j})_{j\in \{3,5,6\}}$. Although $V_{7}$ (wind direction) also exhibits strong dependence with *Y*, we do not consider it here since it is highly correlated with wind speed so would involve adding complex interaction terms to the model formulation, and $V_6$ has a stronger relationship with *Y* compared to $V_7$ (see Fig. 1).

Owing to the complex covariate structure observed in the data, as described in Section 3.1, we employ the flexible EVGAM framework proposed in Youngman (2019) for modelling tail behaviour. Under this framework, GAM formulations are used to capture non-stationarity in the threshold, scale and shape functions given in Eq. 3.3. Without loss of generality, consider the scale function $\sigma (\cdot )$. We assume that

3.4 $$\begin{aligned} h(\sigma (\varvec{\tilde{x}})) = \psi _\sigma (\varvec{\tilde{x}}), \quad \text {with} \quad \psi _{\sigma }(\varvec{\tilde{x}}) = \beta _0 + \sum \limits _{\kappa =1}^{K}\sum \limits _{p=1}^{P_{\kappa }} \beta _{\kappa p}b_{\kappa p} (\varvec{\tilde{x}}), \end{aligned}$$

where $h(x) := \log (x)$ denotes the link function which ensures the correct support, with coefficients $\beta _0,\beta _{\kappa p} \in \mathbb {R}$ and basis functions $b_{\kappa p}$ for $p \in \{1, \ldots , P_{\kappa }\}, \kappa \in \{1, \ldots , K\}$, where *K* is the number of splines in the GAM formulation and $P_{\kappa }$ is the basis dimension relating to spline $\kappa$. The basis functions can be in terms of individual covariates, i.e., $b_{\kappa p}:\mathbb {R}\mapsto \mathbb {R}$, or multiple covariates, i.e., $b_{\kappa p}:\mathbb {R}^m \mapsto \mathbb {R}$, $1 < m \le 8$. Analogous forms can be taken for $v(\cdot )$ and $\xi (\cdot )$, adjusting the link function $h(\cdot )$ as appropriate, although these are not considered here for reasons detailed below.

To select an appropriate threshold, we employ the threshold selection method of Murphy et al. (2024) and extend this approach to select a threshold for non-stationary, covariate-dependent GPD models. The method selects a threshold based on minimising the expected quantile discrepancy (EQD) between the sample quantiles and fitted GPD model quantiles. When fitting a non-stationary model, the excesses will not be identically distributed across covariates. Thus, to utilise the EQD method in this case, we use the fitted non-stationary GPD parameter estimates to transform the excesses to common standard exponential margins and compare sample quantiles against theoretical quantiles from the standard exponential distribution. This transformation is a common approach for checking the model fit of a non-stationary GPD (Coles 2001).

We use a stepped-threshold according to season as there is clear variation in the distribution, and thereby the extremes, of *Y* between seasons; see the Supplementary Material for more details. Specifically, we set $v(\tilde{\varvec{x}}_t):= \mathbbm {1}(\tilde{x}_{2,t} = 1)v_1 + \mathbbm {1}(\tilde{x}_{2,t} = 2)v_2$, $v_1,v_2 \in \mathbb {R}$, with corresponding rate parameter $\zeta (\tilde{\varvec{x}}_t):= \mathbbm {1}(\tilde{x}_{2,t} = 1)\zeta _1 + \mathbbm {1}(\tilde{x}_{2,t} = 2)\zeta _2$, where $\zeta _1, \zeta _2 \in [0,1]$ denote the probabilities of exceeding the threshold for seasons 1 and 2, respectively, and $\tilde{x}_{r,t}$ are realisations of the $r^{\text {th}}$ component of $\tilde{\varvec{x}}$ for $r \in \{1,2,3\}$. This seasonal threshold significantly improves model fits; see the Supplementary Material for further details. GAM forms for the threshold were also explored, but did not offer significant improvement. Furthermore, the smooth GAM formulation of the GPD scale parameter adequately captures any residual variation in the response arising due to covariate dependence.

#### Inference

For all GAM formulations, we only consider basis functions of singular covariates, since specifying basis functions of multiple variables requires a detailed understanding of covariate interactions and can significantly increase the computational complexity of the modelling procedure (Wood 2017). We keep the shape function $\xi (\varvec{x}):=\xi \in \mathbb {R}$ constant across covariates; this is common in non-stationary analyses, since this parameter is difficult to estimate (Chavez-Demoulin and Davison 2005). Within the GAM formulation, we consider several parametric forms to account for the predictive covariates in the scale parameter using linear models, indicator functions and splines.

When using splines, we are required to select a basis dimension $P_{\kappa } \in \mathbb {N}$; this determines the number of coefficients to be estimated. Basis dimension is the most important choice within spline modelling procedures and directly corresponds with the flexibility of the framework (Wood 2017). We only consider splines for $V_3$ and $V_6$. For each $\tilde{X}_{r}$, $r \in \{1,3\}$, we determine the basis dimension $P_{1}$ and $P_{2}$, respectively, by first building a model for $Y_t \mid \tilde{X}_{r,t}$, to allow us to consider the effect of this predictor on the response directly. We vary the basis dimension and compare the resulting models using cross validation (CV), detailed in the following section. We set $P_{1}=4$ and $P_{2}=3$ for $V_3$ and $V_6$, respectively.

For C2, we incorporate the loss function of Eq. 3.2 into the estimation procedure. Let $\mathscr {I}_v := \{ t \in \{1,\ldots ,n\} \mid y_t > v(\tilde{\varvec{x}}_t) \}$ denote the set of temporal indices corresponding to threshold exceedances and $n_v := | \mathscr {I}_v |$. We consider the objective function

3.5 $$\begin{aligned} S(\varvec{\theta }) := - l_R(\varvec{\theta }) + \sum \limits _{i\in \mathscr {I}_v} \mathscr {L}(q_i^*, \hat{q}_i)/n_v, \end{aligned}$$

where $l_R(\varvec{\theta })$ denotes the penalised log-likelihood function of the restricted maximum likelihood estimation (REML) approach (Wood 2017), $\varvec{\theta }$ denotes the parameter vector associated with the GPD formulation of Eq. 3.4, and $\sum _{i\in \mathscr {I}_v} \mathscr {L}(q_i^*, \hat{q}_i)/n_v$ denotes the average loss between the sample quantiles of the transformed excesses and the theoretical standard exponential quantiles. Specifically, we transform the excesses, $(y_{t} - v(\tilde{\varvec{x}}_t))_{t \in \mathscr {I}_v}$, to standard exponential margins using the fitted non-stationary GPD parameter estimates and compare the ordered excesses, $\varvec{q}^*$, to the theoretical quantiles, $\hat{\varvec{q}}$, from a standard exponential distribution evaluated at probabilities $\{p_i = i/(n_v + 1), i=1, \ldots , n_v\}$. Minimising the objective function $S(\varvec{\theta })$ ensures that the parameter estimates also account for and minimise the loss function, $\mathscr {L}$. We use this formulation to adjust the GPD parameters for challenge C2 once a threshold is selected.

#### Model selection

To determine the best-fitting model, we use a forward selection process and aim to minimise the model’s CV score. For each model, we apply *k*-fold CV (Hastie et al. 2001, Ch 7.) utilising the continuous ranked probability score (CRPS, Gneiting and Katzfuss 2014) as our goodness-of-fit metric. CRPS describes the discrepancy between the predicted distribution function and observed values without the specification of empirical quantiles. We explore model ranking by taking both $k = 10$ and 50, and find that both give an equivalent ranking; we present results for the latter. We also provide the Akaike Information Criterion (AIC) and Bayesian Information Criterion (BIC) values to aid in model selection. A subset of models used in the forward selection process are detailed in Table 1 where, for each model, we provide the change in the CRPS, AIC and BIC relative to model 1. The parameterisation of model 7 achieves the largest reduction for all three metrics relative to the baseline model.

**Table 1:** Table of selected models considered for challenge C1

Model	$\sigma (\tilde{\varvec{x}}_{t})$	$\Delta$CRPS	$\Delta$AIC	$\Delta$BIC
1	$\beta _{0}$	0	0	0
2	$\beta _{0} + \beta _{1} \mathbbm {1}(\tilde{x}_{2,t} = 1)$	−0.5	−33.4	−26.1
3	$\beta _{0} + s_{1}(\tilde{x}_{1,t})$	−0.9	−408.5	−379.2
4	$\beta _{0} + s_{2}(\tilde{x}_{3,t})$	−0.5	−284.3	−276.8
5	$\beta _{0} + \beta _{1} \mathbbm {1}(\tilde{x}_{2,t} = 1) + s_{1}(\tilde{x}_{1,t})$	−0.9	−425.8	−388.1
6	$\beta _{0} + s_{1}(\tilde{x}_{1,t}) + s_{2}(\tilde{x}_{3,t})$	−1.0	−752.7	−717.2
7	$\beta _{0} + \beta _{1} \mathbbm {1}(\tilde{x}_{2,t} = 1) + s_{1}(\tilde{x}_{1,t}) + s_{2}(\tilde{x}_{3,t})$	$\mathbf {-1.1}$	$\mathbf {-780.0}$	$\mathbf {-735.3}$

$\mathbbm {1}(\cdot )$ denotes an indicator function, $s_{i}(\cdot )$ for $i \in \{1,2\}$ denote thin-plate regression splines, $\beta _0,\beta _1$ are coefficients to be estimated, and $\tilde{{x}}_{r,t}$ is defined as in the text. All values have been given to one decimal place

Numbers in bold highlight the smallest values in each case and indicate the largest change compared to the baseline model for each of the model selection metrics

### Uncertainty

For each of the 100 different covariate combinations, $\tilde{\varvec{x}}_i$ for $i\in \{1,\ldots ,100\}$, we need to construct central 50% confidence intervals. We use a bootstrapping procedure to avoid making potentially inaccurate assumptions such as the asymptotic normality approximation of maximum likelihood estimates, for example. Traditional bootstrap approaches are non-parametric and randomly resample the data with replacement. However, in Section 3.1 we find that the response variable is dependent on covariates, and these covariates exhibit temporal dependence. A standard bootstrap procedure would therefore not retain this dependence. Instead, we preserve the temporal dependence structure of covariates and their relationship with the response variable by approximating our confidence intervals using the stationary, semi-parametric bootstrapping procedure adopted by D’Arcy et al. (2023).

First, the response variable $Y_t$ is transformed to Uniform(0,1) margins to preserve its non-stationary behaviour; denote this sequence $U_{t}^{Y}=F_{{Y}_{t}|{\tilde{\varvec{X}}}_{t}}(Y_{t}|\tilde{\varvec{X}}_{t}=\tilde{x}_{t})$ where $F_{Y_t|\tilde{\varvec{X}}_t}$ is the estimated model given in Eq. 3.3. We then adopt the stationary bootstrap procedure of Politis and Romano (1994) to retain the temporal dependence in the response and explanatory variables by sampling blocks of consecutive observations. The block length *L* is random and simulated from a Geometric(1/*l*) distribution, where the mean block length $l\in \mathbb {N}$ is carefully selected based on the autocorrelation function. This was selected at 50 days, the maximum lag for which the autocorrelation was significant across all variables; see the Supplementary Material. Denote this bootstrapped sequence on Uniform margins by $U_t^B$. We transform $U_t^B$ back to the original scale using our fitted model, preserving the original structure of $Y_t$; we denote this series $Y_t^B$. Then we fit our model to $Y_t^B$ to re-estimate all of the parameters and thus the quantile of interest. We repeat this procedure to obtain 200 bootstrap samples.

### Results

**Fig. 2:**
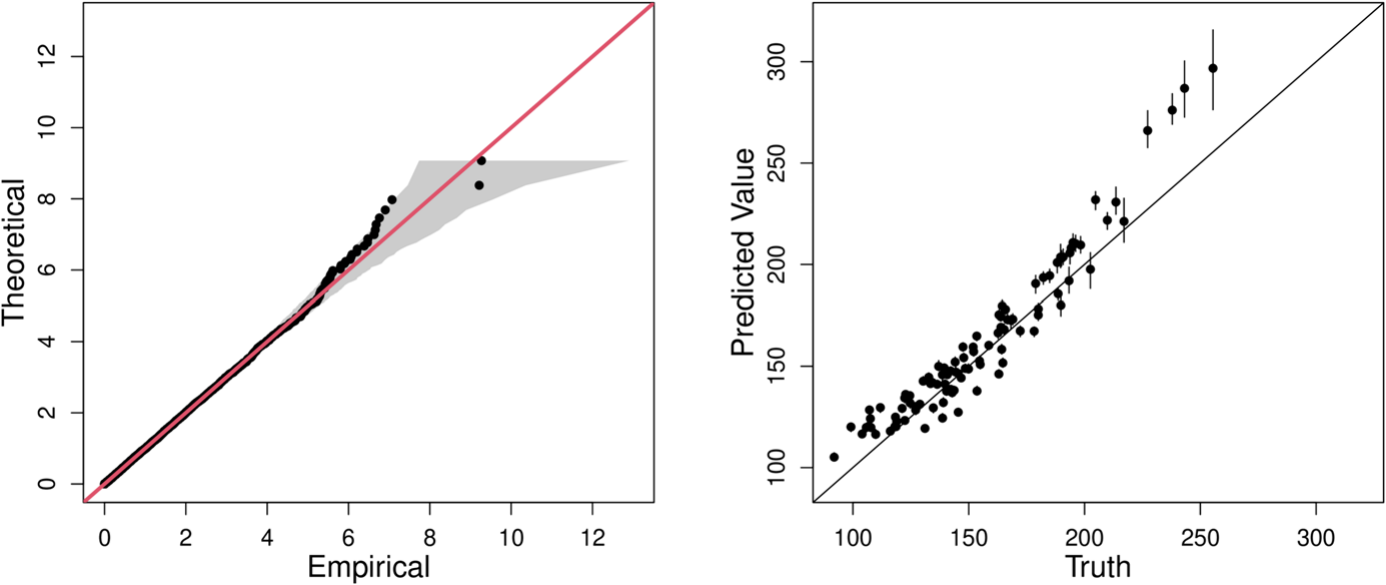
QQ plot for our final model (model 7 in Table 1) on standard exponential margins. The $y=x$ line is given in red and the grey region represents the 95% tolerance bounds (left). Predicted $0.9999-$quantiles against true quantiles for the 100 covariate combinations. The points are the median predicted quantile over 200 bootstrapped samples and the vertical error bars are the corresponding 50% confidence intervals. The $y = x$ line is also shown (right)

For C1, we use our final model of Section 3.2.3 to estimate the 0.9999-quantile of $Y\mid \tilde{\varvec{X}} = \tilde{\varvec{x}}_{i}$, $i \in \{1,\ldots ,100\}$, for the set of 100 covariate combinations. The left panel of Fig. 2 shows the quantile-quantile (QQ) plot for our model. There is general alignment between the model and empirical quantiles; however, there is some over-estimation in the upper tail, and our 95% tolerance bounds do not contain some of the most extreme response values. The right panel of Fig. 2 shows our predicted quantiles, and their associated confidence intervals, compared to their true quantiles. As expected, our predictions tend to over-estimate the true quantiles. We note this figure is different from the one presented by Rohrbeck et al. (2023) due to an error in our code being fixed after submission. In this scenario, our estimated confidence intervals lead to a $14\%$ coverage of the true quantiles, which does not alter our ranking for this challenge. Our performance and model improvements are discussed in Section 6.

For challenge C2, we estimate the quantile of interest as $\hat{q} = 213.1 \; (209.3, 242.1)$. A 95% confidence interval for the estimate is given in parentheses based on the bootstrapping procedure outlined in Section 3.2.1. Due to a coding error, this value differs from the original estimate submitted for the EVA (2023) Conference Data Challenge. The updated value over-estimates compared to the truth ($q=196.6$).

## Challenge C3

### Exploratory data analysis

For challenge C3, we are provided with 70 years of daily data of an environmental variable for three towns on the island of Coputopia. These data are denoted by $Y_{i,t}$, $i \in \{1,2,3\}$, $t \in \{1,\ldots ,n\}$, where *i* is the index of each town and *t* is the point in time. Each year consists of 12 months, each lasting 25 days, resulting in $n=21,000$ observations for each location.

We are also provided with daily covariate observations $\varvec{X}_t = (S_t,A_t)$, where $S_t$ and $A_t$ denote seasonal and atmospheric conditions, respectively. Season is a binary variable, taking values in the set $\{1,2\}$, with each year of observations exhibiting both seasons for exactly 150 consecutive days. Atmospheric conditions are piecewise constant over months, with large variation in the observed values between months. A descriptive figure of both covariates is given in the Supplementary Material.

In Rohrbeck et al. (2023), we are informed that $Y_{i,t}$ are distributed identically across all sites and over time, with standard Gumbel margins. However, it is not known whether the covariates $\varvec{X}_t$ influence the dependence structure of $\varvec{Y}_t := (Y_{1,t},Y_{2,t},Y_{3,t})$. We are also informed that, conditioned on covariates, the process is independent over time, i.e., $(\varvec{Y}_{t} \mid \varvec{X}_t)\perp \!\!\!\!\perp (\varvec{Y}_{t'} \mid \varvec{X}_{t'})$ for any $t \ne t'$. In this section, we examine what influence, if any, the covariate process $\varvec{X}_t$ may have on the dependence structure of $\varvec{Y}_t$.

We begin by transforming the time series $Y_{i,t}$ to standard exponential margins, denoted by $\varvec{Z}_{i,t}$, via the probability integral transform. This transformation is common in the study of multivariate extremes and can simplify the description of extremal dependence (Keef et al. 2013a). To explore the extremal dependence in the Coputopia time series, we consider all 2- and 3-dimensional subvectors of the process, i.e., $\{ Z_{i,t}, i \in I, t \in \{1, \ldots , n\}\}, \; I \in \mathscr {I}:= \{ \{1,2\}, \{ 1,3\} , \{ 2,3\} , \{ 1,2,3\} \}.$ This separation is important to ensure the overall dependence structure is fully understood, since intermediate scenarios can exist where a random vector exhibits $\chi =0$, but $\chi >0$ for some 2-dimensional subvector(s) (Simpson et al. 2020).

Furthermore, to explore the impact of covariates on the dependence structure, we partition the time series into subsets using the covariates. For the seasonal covariate, let $G^S_{I,j} := \{ Z_{i,t}, i \in I, S_t = j \}$ for $j = 1,2,$ and for the atmospheric covariate, let $\pi : \{1,\ldots ,n\} \rightarrow \{1,\ldots ,n\}$ denote the permutation associated with the order statistics of $A_t$, defined so that ties in the data are accounted for. We then split the data into 10 equally sized subsets corresponding to the atmospheric order statistics, i.e., $G^A_{I,k} := \left\{ Z_{i,t}, i \in I, t \in \Sigma ^k \right\}$ for $k = 1,2,\ldots ,10,$ where $\Sigma ^k := \{ t \mid (k-1)n/10 + 1 \le \pi (t) \le kn/10 \}$. Thus, the atmospheric values associated with each subset $G^A_{I,k}$ will increase over *k*.

**Fig. 3:**
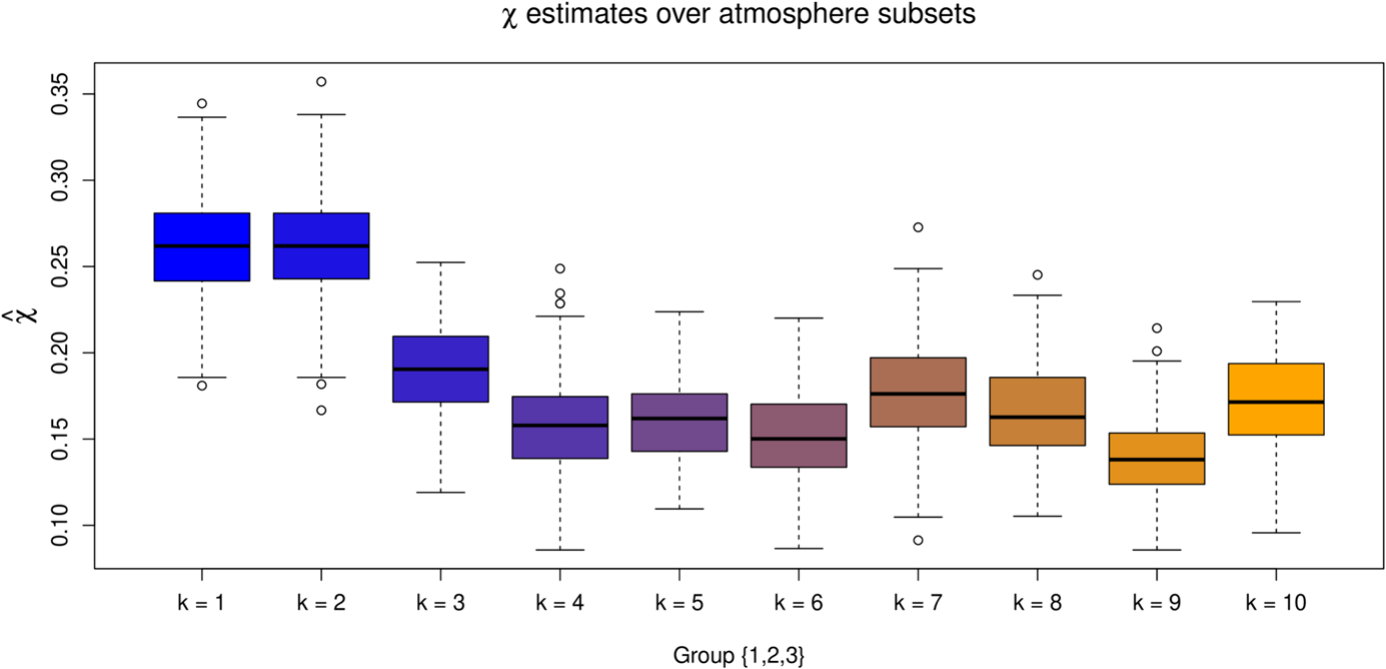
Boxplots of empirical $\chi$ estimates obtained for the subsets $G^A_{I,k}$, with $k = 1, \ldots , 10$ and $I=\{1,2,3\}$. The colour transition (from blue to orange) over *k* illustrates the trend in $\chi$ estimates as the atmospheric values are increased

The idea behind these subsets is to examine whether altering the values of either covariate impacts the extremal dependence structure. Consequently, we set $u=0.9$ and estimate $\chi (u)$ using the techniques outlined in Section 2, with uncertainty quantified through bootstrapping with 200 samples. The bootstrapped $\chi$ estimates for $G^A_{I,k}$ with $I = \{1,2,3\}$ are given in Fig. 3. The plots for the remaining index sets in $\mathscr {I}$, along with the subsets associated with the seasonal covariate, are given in the Supplementary Material. The estimates of $\chi$ appear to vary, in the majority of cases, across both subset types (seasonal and atmospheric), suggesting both covariates have an impact on the dependence structure. For the atmospheric process in particular, the values of $\chi$ tend to decrease for higher atmospheric values, suggesting a negative association between the strength of positive extremal dependence and the atmospheric covariate. We also observe that across all subsets, $\chi$ appears consistently low in magnitude, suggesting the extremes of some, if not all, of the sub-vectors are unlikely to occur simultaneously. As such, for modelling the Coputopia time series, we require a framework that can capture such forms of dependence. We also consider pointwise estimates of the function $\lambda (\cdot )$, as defined later in Eq. 4.1, over $G^S_{I,j}$ and $G^A_{I,k}$ for fixed simplex points; these results are given in the Supplementary Material. Similar to $\chi$, estimates of $\lambda (\cdot )$ vary significantly across subsets, providing additional evidence of non-stationarity within extremal dependence structure.

### Modelling of joint tail probabilities under asymptotic independence

For challenge C3, we are required to estimate probabilities $$p_1:=\Pr \left( Y_1>y, Y_2>y,\right. $$$$\left. Y_3>y\right) $$ and $$p_2:=\Pr \left( Y_1>v, Y_2>v, Y_3<m\right) $$, with $$y=6$$, $$v = 7$$ and $$m = -\log (\log (2))$$. Note that $$p_1$$ and $$p_2$$ are independent of the covariate process and correspond to different extremal regions in $$\mathbb {R}^3$$; we refer to $$p_1$$ and $$p_2$$ as parts 1 and 2 of the challenge, respectively. For the remainder of this section we will consider the transformed exponential variables $$(Z_1,Z_2,Z_3)$$, omitting the subscript *t* for ease of notation. Observe that $$F_{(-Z_3)}(z)=e^z,$$ for $$z<0;$$ setting $$\tilde{Z}_3 := -\log \left( 1-\exp (-Z_3)\right) ,$$ we have

$$\begin{aligned} p_2 = \Pr \left( Z_1>\tilde{v}, Z_2>\tilde{v}, Z_3<\tilde{m}\right) =\Pr \left( Z_1>\tilde{v}, Z_2>\tilde{v}, \tilde{Z}_3>\tilde{m}\right) , \end{aligned}$$

where $\tilde{v}$ and $\tilde{m}$ denote the values *v* and *m* transformed to the standard exponential scale, e.g., $\tilde{v}:=-\log \left( 1-\exp (-\exp (-v))\right)$. Similarly, we have $p_1 = \Pr \left( Z_1>\tilde{y}, Z_2>\tilde{y}, Z_3>\tilde{y}\right)$. Consequently, both $p_1$ and $p_2$ can be considered as joint survivor probabilities.

Since not all extremes of $Z_1$, $Z_2$ and $Z_3$ are observed simultaneously, we employ the framework by Wadsworth and Tawn (2013), which is a generalisation of the approach proposed in Ledford and Tawn (1996). The model of Wadsworth and Tawn (2013) assumes that for any ray $\varvec{\omega }\in \varvec{S}^2:= \left\{ (\omega _1,\omega _2,\omega _3)\in [0,1]^3: \omega _1+\omega _2 +\omega _3=1\right\} ,$ where $\varvec{S}^2$ denotes the standard 2-dimensional simplex,

4.1 $$\begin{aligned} \begin{array}{rcl} \text {Pr}\left( Z_1>\omega _1r,\,Z_2>\omega _2r,\, Z_3>\omega _3r\right) & =& \text {Pr}\left( \min \{Z_1/\omega _1,\,Z_2/\omega _2,\, Z_3/\omega _3\}>r\right) \\ & =& \mathscr {L}(e^r;\varvec{\omega })e^{-r\lambda (\varvec{\omega })}, \end{array} \end{aligned}$$

as $r \rightarrow \infty$, where $\lambda (\varvec{\omega }) \ge \max (\varvec{\omega })$ is known as the angular dependence function (ADF). Asymptotic dependence occurs at the lower bound, i.e., $\lambda (\varvec{\omega }) = \max (\varvec{\omega })$ for all $\varvec{\omega } \in \varvec{S}^2$, and the coefficient of tail dependence is related to the ADF via $\eta = 1/\{3\lambda (1/3,1/3,1/3)\}$. In practice, Eq. 4.1 can be used to evaluate extreme joint survivor probabilities; in particular, probabilities $p_1$ and $p_2$ can be identified with the rays $\varvec{\omega }^{(1)}:=(\tilde{y}, \tilde{y},\tilde{y})/r^{(1)}$ and $\varvec{\omega }^{(2)}:=(\tilde{v}, \tilde{v},\tilde{m})/r^{(2)}$ in $\varvec{S}^2$, respectively, where $r^{(1)} := \tilde{y}+\tilde{y}+\tilde{y}$ and $r^{(2)} := \tilde{v}+\tilde{v}+\tilde{m}$. See Section 4.4 for further details.

### Accounting for non-stationary dependence

In the stationary setting, pointwise estimates of $\lambda (\cdot )$ can be obtained via the Hill estimator (Hill 1975), from which tail probabilities can be approximated. However, alternative procedures are required for data exhibiting trends in dependence, such as the Coputopia data set. Existing approaches for capturing non-stationary dependence structures are sparse in the extremes literature, and most approaches are limited to asymptotically dependent data structures. For the case when data are not asymptotically dependent, Mhalla et al. (2019) and Murphy-Barltrop and Wadsworth (2024) propose non-stationary extensions of the Wadsworth and Tawn (2013) framework, while Jonathan et al. (2014) and Guerrero et al. (2023) propose non-stationary extensions of the Heffernan and Tawn (2004) model (see Murphy-Barltrop and Wadsworth 2024 for a detailed review).

To account for non-stationary dependence in C3, we propose an extension of the Wadsworth and Tawn (2013) framework. With $\varvec{Z}_t = (Z_{1,t},Z_{2,t},Z_{3,t})$ and $\varvec{X}_t$, defined as in Section 4.1, we define the structure variable $T_{\varvec{\omega },t} := \min \{Z_{1,t}/\omega _1,Z_{2,t}/\omega _2,Z_{3,t}/\omega _3\}$, for any $\varvec{\omega } \in \varvec{S}^2$; we refer to $T_{\varvec{\omega },t}$ as the min-projection variable at time *t*. From Section 4.1, we know that the joint distribution of $\varvec{Z}_t$ is not identically distributed over *t*; which implies non-stationarity in the distribution of $T_{\varvec{\omega },t}$. To account for this, Mhalla et al. (2019) and Murphy-Barltrop and Wadsworth (2024) assume the following model given the vector of covariates $\varvec{x}_t$:

4.2 $$\begin{aligned} \operatorname {Pr}\left( T_{\varvec{\omega },t}>u \mid \varvec{X}_t=\varvec{x}_t\right) =\mathscr {L}\left( e^u \mid \varvec{\omega }, \varvec{x}_t\right) e^{-\lambda \left( \varvec{\omega }; \varvec{x}_t\right) u} \text{ as } u \rightarrow \infty , \end{aligned}$$

for all *t*, where $\lambda \left( \cdot ; \varvec{x}_t\right)$ denotes the non-stationary ADF. Note that this assumption is very similar in form to Eq. 4.1, with the primary difference being the function $\lambda (\cdot ; \varvec{x}_t)$ is non-stationary over *t*. From Eq. 4.2, we have

4.3 $$\begin{aligned} \operatorname {Pr}\left( T_{\varvec{\omega },t}-u>z \mid T_{\varvec{\omega },t}>u, \varvec{X}_t=\varvec{x}_t\right) = e^{-\lambda \left( \varvec{\omega } ; \varvec{x}_t\right) z} \text{ as } u \rightarrow \infty , \end{aligned}$$

for $z>0$. Consequently, Eq. 4.2 is equivalent to assuming $(T_{\varvec{\omega },t} - u) \mid \{ T_{\varvec{\omega },t} > u,\varvec{X}_t=\varvec{x}_t\} \sim \text {Exp}(\lambda \left( \varvec{\omega } ; \varvec{x}_t\right) )$ as $u\rightarrow \infty$.

We found that (4.2) was not flexible enough to capture the tail of $T_{\varvec{\omega },t}$ for the Coputopia data; see Section 4.3.2 for further discussion. Thus, we propose the following model: given any $z>0$ and a fixed $\varvec{\omega } \in \varvec{S}^2$, we assume

4.4 $$\begin{aligned} \operatorname {Pr}\left( T_{\varvec{\omega },t}-u>z \mid T_{\varvec{\omega },t}>u, \varvec{X}_t=\varvec{x}_t\right) = \left( 1 + \frac{\xi \left( \varvec{\omega }; \varvec{x}_t\right) z}{\sigma \left( \varvec{\omega }; \varvec{x}_t\right) } \right) ^{-1/\xi \left( \varvec{\omega }; \varvec{x}_t\right) } \text{ as } u \rightarrow \infty , \end{aligned}$$

where $\sigma (\cdot ; \varvec{x}_t), \xi (\cdot ; \varvec{x}_t)$ are non-stationary scale and shape parameter functions, respectively. This is equivalent to assuming $(T_{\varvec{\omega },t} - u) \mid \{ T_{\varvec{\omega },t} > u,\varvec{X}_t=\varvec{x}_t\} \sim \text {GPD}(\sigma \left( \varvec{\omega }; \varvec{x}_t\right) ,\,\xi \left( \varvec{\omega }; \varvec{x}_t\right) )$ as $u\rightarrow \infty$, and Eq. 4.3 is recovered by taking the limit as $\xi \left( \varvec{\omega }; \varvec{x}_t\right) \rightarrow 0$ for all *t*.

Our proposed formulation in Eq. 4.4 allows for additional flexibility within the modelling framework by including a GPD shape parameter $\xi \left( \varvec{\omega };\varvec{x}_t\right)$, which quantifies the tail behaviour of $T_{\varvec{\omega },t}$. Given the wide range of distributions in the domain of attraction of a GPD (Pickands 1975), it is reasonable to assume that the tail of $T_{\varvec{\omega },t}$ can be approximated by Eq. 4.4. For the Coputopia time series, this assumption appears valid, as demonstrated by the diagnostics in Section 4.3.2.

#### Model fitting

To apply (4.4), we first fix $\varvec{\omega } \in \varvec{S}^2$ and assume that the formulation holds approximately for some sufficiently high threshold level from the distribution of $T_{\varvec{\omega },t}$; we denote the corresponding quantile level by $\tau \in (0,1)$. For simplicity, the same quantile level is considered across all *t*. Further, let $v_{\tau }(\varvec{\omega },\varvec{x}_t)$ denote the corresponding threshold function, i.e., $\Pr (T_{\varvec{\omega },t} \le v_{\tau }(\varvec{\omega },\varvec{x}_t) \mid \varvec{X}_t=\varvec{x}_t) = \tau$ for all *t*. Under our assumption, we have $(T_{\varvec{\omega },t} - v_{\tau }(\varvec{\omega },\varvec{x}_t)) \mid \{ T_{\varvec{\omega },t} > v_{\tau }(\varvec{\omega },\varvec{x}_t),\varvec{X}_t=\varvec{x}_t\} \sim \text {GPD}(\sigma \left( \varvec{\omega }; \varvec{x}_t\right) ,\xi \left( \varvec{\omega }; \varvec{x}_t\right) )$. We emphasise that $v_{\tau }(\varvec{\omega },\varvec{x}_t)$ is not constant in *t*, and we would generally expect $v_{\tau }(\varvec{\omega },\varvec{x}_t) \ne v_{\tau }(\varvec{\omega },\varvec{x}_{t'})$ for $t \ne t'$.

As detailed in Section 4.2, both $p_1$ and $p_2$ can be associated with points on the simplex $\varvec{S}^2$, denoted by $\varvec{\omega }^{(1)}$ and $\varvec{\omega }^{(2)}$, respectively. Letting $\varvec{\omega } \in \{\varvec{\omega }^{(1)},\varvec{\omega }^{(2)}\}$, our estimation procedure consists of two stages: estimation of the threshold function $v_{\tau }(\varvec{\omega },\varvec{z}_t)$ for a fixed $\tau \in (0,1)$, followed by estimation of GPD parameter functions $\sigma \left( \varvec{\omega }; \varvec{x}_t\right) ,\xi \left( \varvec{\omega }; \varvec{x}_t\right)$. For both steps, we take a similar approach to Section 3.2 and use GAMs to capture these covariate relationships. To simplify our approach, we falsely assume that the atmospheric covariate $A_t$ is continuous over *t*; this step allows us to utilise GAM formulations containing smooth basis functions. Given the significant variability in $A_t$ between months, discrete formulations for this covariate would significantly increase the number of model parameters and result in higher variability.

Let $\log ( v_{\tau }(\varvec{\omega },\varvec{x}_t)) = \psi _v(\varvec{x}_t)$, $\log ( \sigma \left( \varvec{\omega }; \varvec{x}_t\right) ) = \psi _{\sigma }(\varvec{x}_t)$ and $\xi \left( \varvec{\omega }; \varvec{x}_t\right) = \psi _{\xi }(\varvec{x}_t)$ denote the GAM formulations of each function, where $\psi _{-}$ denotes the basis representation of Eq. 3.4. Exact forms of basis functions are specified in Section 4.3.2. As in Section 3.2, model fitting is carried out using the |evgam| software package (Youngman 2022). For the first stage, $v_{\tau }(\varvec{\omega },\varvec{x}_t)$ is estimated by exploiting a link between the loss function typically used for quantile regression and the asymmetric Laplace distribution (Yu and Moyeed 2001). The spline coefficients associated with $\psi _{\sigma }$ and $\psi _{\xi }$ are estimated subsequently using the obtained threshold exceedances.

#### Selection of GAM formulations and diagnostics

Prior to estimation of the threshold and parameter functions, we specify a quantile level $\tau$ and formulations for each of the GAMs. To begin, we fix $\tau =0.9$ and consider a variety of formulations for each $\psi _v, \psi _{\sigma }$ and $\psi _{\xi }$. By comparing metrics for model selection, namely AIC, BIC and CRPS, we found the following formulations to be sufficient

4.5 $$\begin{aligned} \psi _v(\varvec{x}_t) = \beta _u + s_v(a_t) + \beta _s \mathbbm {1}(s_t = 2), \quad \psi _{\sigma }(\varvec{x}_t) = \beta _{\sigma } + s_{\sigma }(a_t)\quad \text {and}\quad \psi _{\xi }(\varvec{x}_t) = \beta _{\xi }, \end{aligned}$$

for parts 1 and 2, where $\beta _{u}, \beta _{\sigma }, \beta _{\xi } \in \mathbb {R}$ denote constant intercept terms, $\mathbbm {1}$ denotes the indicator function with corresponding coefficient $\beta _s \in \mathbb {R}$, and $s_{u},s_{\sigma }$ denote cubic regression splines of dimension 10. The shape parameter is set to constant for the reasons outlined in Section 3.2.3. Cubic basis functions are used for $\psi _v$ and $\psi _{\sigma }$ since they have several desirable properties, including continuity and smoothness (Wood 2017). A dimension of size 10 appears more than sufficient to capture the trends relating to the atmosphere variable. Alternative formulations were tested for both parts, but this made little difference to the resulting model fits.

We remark that the seasonal covariate is only present with the formulation for $\psi _v$. Once accounted for in the non-stationary threshold, the seasonal covariate appeared to have little influence on the fitted GPD parameters. More complex GAM formulations were tested involving interaction terms between the seasonal and atmospheric covariates, which showed little to no improvement in model fits. Thus, we prefer the simpler formulations on the basis of parsimony.

With GAM formulations selected, we now consider the quantile level $\tau \in (0,1)$. To assess sensitivity in our formulation, we set $\textrm{T}:= \{0.8,0.81,\ldots ,0.99\}$ and fit the GAMs outlined in Eq. 4.5 for each $\tau \in \textrm{T}$. Letting $\delta _{\varvec{\omega },t}$ and $\mathscr {T}_{\tau }:=\{ t \in \{1,\ldots ,n\} \mid \delta _{\varvec{\omega },t} > v_{\tau }(\varvec{\omega },\varvec{x}_t)\}$ denote the min-projection observations and indices of threshold-exceeding observations, respectively, we expect the set $\mathscr {E}:=\{ -\log \left\{ 1 - F_{GPD}( \delta _{\varvec{\omega },t} - v_{\tau }(\varvec{\omega },\varvec{x}_t)) \mid \sigma \left( \varvec{\omega }; \varvec{x}_t\right) ,\xi \left( \varvec{\omega };\varvec{x}_t\right) \right\} \mid t \in \mathscr {T}_{\tau }\}$ to follow a standard exponential distribution.

**Fig. 4:**
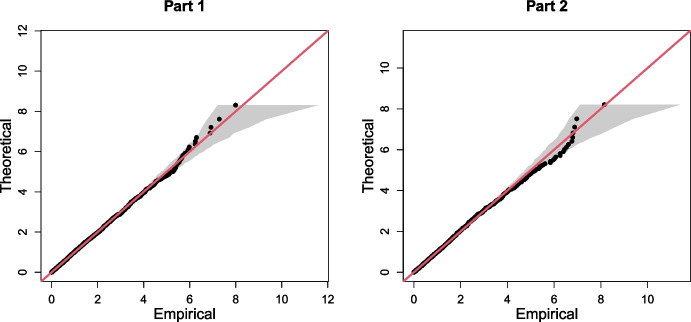
Final QQ plots for parts 1 (left) and 2 (right) of C3, with the $y=x$ line given in red. In both cases, the grey regions represent the 95% bootstrapped tolerance bounds

With all exceedances transformed to a unified scale, we compare the empirical and model exponential quantiles using QQ plots, through which we assess the relative performance of each $\tau \in \textrm{T}$. We selected $\tau$ values for which the empirical and theoretical quantiles appeared most similar in magnitude. From this analysis, we set $\tau = 0.83$ and $\tau = 0.85$ for parts 1 and 2, respectively. The corresponding QQ plots are given in Fig. 4, where we observe reasonable agreement between the empirical and theoretical quantiles. However, whilst these values appeared optimal within $\textrm{T}$, we stress that adequate model fits were also obtained for other quantile levels, suggesting our modelling procedure is not particularly sensitive to the exact choice of quantile. Furthermore, we also tested a range of quantile levels below the 0.8-level, but were unable to improve the quality of model fits.

Plots illustrating the estimated GPD scale parameter functions are given in the Supplementary Material, with the resulting dependence trends in agreement with the observed trends from Section 4.1. We also remark that the estimated GPD shape parameters obtained for parts 1 and 2 were $0.042\, (0.01,0.075)$ and $0.094\,(0.059,0.128)$, respectively, where the brackets denote $95\%$ confidence intervals obtained using posterior sampling (Wood 2017). These estimates, which indicate slightly heavy-tailed behaviour within the min-projection variable, provide insight into why the original exponential modelling framework is not appropriate for C3.

Overall, these results suggest different extremal dependence trends exist for the two simplex points $\varvec{\omega }^{(1)}$ and $\varvec{\omega }^{(2)}$, illustrating the importance of the flexibility in our model. These findings are also in agreement with empirical trends observed in Section 4.1, suggesting our modelling framework is successfully capturing the underlying extremal dependence structures.

### Results

Given estimates of threshold and parameter functions, probability estimates can be obtained via Monte Carlo techniques. Taking $p_1$, for instance, we have

$$\begin{aligned} \begin{array}{rcl} p_1 & =& \Pr (Z_1>\tilde{y},Z_2>\tilde{y},Z_3>\tilde{y}) \\ & =& \Pr \left( \min \left( Z_1/\omega ^{(1)}_1, Z_2/\omega ^{(1)}_2, Z_3/\omega ^{(1)}_3 \right)> r^{(1)}\right) \\ & =& \displaystyle {\int _{\varvec{X}_t} \Pr \left( T_{\varvec{\omega }^{(1)},\;t}> r^{(1)} \mid \varvec{X}_t = \varvec{x}_t\right) f_{\varvec{X}_t}(\varvec{x}_t) \textrm{d}\varvec{x}_t} \\ & =& \displaystyle {(1-\tau )\int _{\varvec{X}_t} \Pr (T_{\varvec{\omega }^{(1)},\;t}> r^{(1)} \mid T_{\varvec{\omega }^{(1)},\;t} > v_{\tau }(\varvec{\omega }^{(1)},\varvec{x}_t),\varvec{X}_t = \varvec{x}_t) f_{\varvec{X}_t}(\varvec{x}_t) \textrm{d}\varvec{x}_t} \\ & \approx & \displaystyle {\frac{1-\tau }{n} \sum _{t=1}^n \left( 1 + \frac{\xi (\varvec{\omega }^{(1)}; \varvec{x}_t) \left( r^{(1)} - v_{\tau }(\varvec{\omega }^{(1)},\varvec{x}_t)\right) }{\sigma \left( \varvec{\omega }^{(1)}; \varvec{x}_t\right) } \right) ^{-1/\xi \left( \varvec{\omega }^{(1)}; \varvec{x}_t\right) }}, \end{array} \end{aligned}$$

assuming $\{ \varvec{x}_t: t \in \{1,\ldots ,n\}\}$ is a representative sample from $\varvec{X}_t$. The procedure for $p_2$ is analogous. We note that this estimation procedure is only valid when $r^{(1)} > v_{\tau }(\varvec{\omega }^{(1)},\varvec{x}_t)$, or $r^{(2)} > v_{\tau }(\varvec{\omega }^{(2)},\varvec{x}_t)$, for all *t*: however, for each $\tau \in \textrm{T}$, this inequality is always satisfied, owing to the very extreme nature of the probabilities in question. Through this approximation, we obtain $\hat{p}_1=1.480\times 10^{-5}$ and $\hat{p}_2=2.461\times 10^{-5}.$

## Challenge C4

### Exploratory data analysis

Challenge C4 entails estimating survival probabilities across 50 locations on the island of Utopula. As stated in Rohrbeck et al. (2023), the Utopula island is split in two administrative areas, for which the respective regional governments 1 and 2 have collected data concerning the variables $Y_{i,t}$, $i \in I = \{1,\ldots ,50\},\, t \in \{1,\ldots ,10,000$}. Index *i* denotes the $i^{\text {th}}$ location, with locations $i \in \{1, \ldots ,25\}$ and $i \in \{26, \ldots ,50\}$ belonging to the administrative areas of governments 1 and 2, respectively. Index *t* denotes the time point in days; however, since $Y_{i,t}$ are IID for all *i*, we drop the subscript *t* for the remainder of this section.

**Fig. 5:**
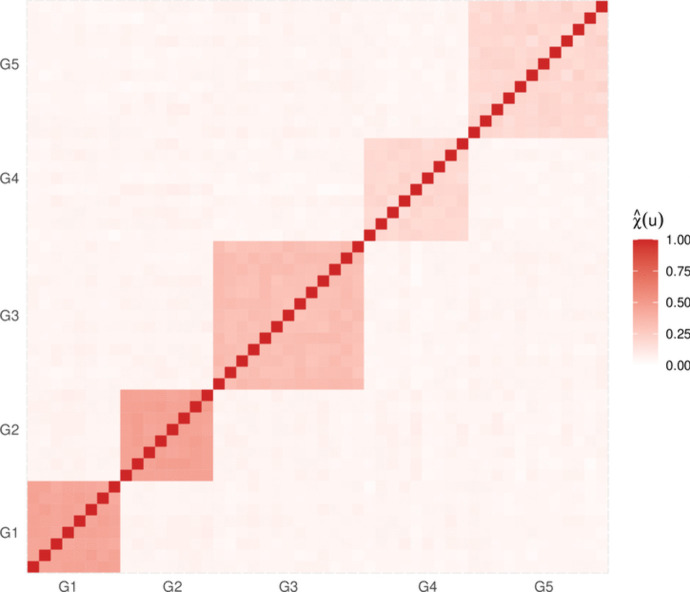
Heat map of estimated empirical pairwise $\chi (u)$ extremal dependence coefficients with $u=0.95$

Since many multivariate extreme value models are only applicable in low-to-moderate dimensions, we consider dimension reduction based on an exploration of the extremal dependence structure of the data. In particular, we analyse pairwise estimates of the extremal dependence coefficient $\chi (u)$, introduced in Eq. 2.2, for all possible pairwise combinations of sites; the resulting estimates, using $u=0.95$, are presented in the heat map of Fig. 5. Identification of any dependence clusters is achieved through visual investigation, which seems appropriate for this data. We note, however, that should visual considerations not suffice, alternative more sophisticated clustering methods are available and can be applied; see for example Bernard et al. (2013).

Figure 5 suggests the existence of 5 distinct subgroups where all variables within each subgroup have similar extremal dependence characteristics, while variables in different subgroups appear to be approximately independent of each other in the extremes. It is worth mentioning that the same clusters are identified when we analyse pairwise estimates of the extremal dependence coefficient $\eta (u)$; the resulting estimates can be found in the Supplementary Material. Moreover, examining the magnitudes of $\chi (\cdot )$ and $\eta (\cdot )$ estimates, it does not appear reasonable to assume asymptotic dependence between variables in the same group. We therefore consider models that can be applied to data structures that do not take their extreme values simultaneously. The indices of the five aforementioned subgroups are $G_1=\{4, 14, 19, 28, 30, 38, 43, 44\}$, $G_2=\{3, 10, 15, 18, 22, 29, 45, 47\}$, $G_3=\{8, 21, 25, 26, 32, 33, 34, 40, 41, 42, 48, 49, 50\}$, $G_4=\{1, 2, 5, 7, 9, 17, 20, 31, 46\}$ and $G_5=\{6, 11, 12, 13, 16, 23, 24, 27, 35, 36, 37, 39\}$. Groups $G_1$ and $G_2$ include the most strongly dependent variables (shown by the darkest color blocks in Fig. 5), followed by group $G_3$, while groups $G_4$ and $G_5$ contain the most weakly dependent variables. We henceforth assume independence between these groups of variables, i.e., $\Pr ((Y_i)_{i \in G_k} \in A_k,(Y_i)_{i \in G_{k'}} \in A_{k'}) = \Pr ((Y_i)_{i \in G_k} \in A_k)\Pr ((Y_i)_{i \in G_{k'}} \in A_{k'})$, $A_k\subset \mathbb {R}^{\vert G_k \vert },A_{k'}\subset \mathbb {R}^{\vert G_{k'} \vert }$, for any $k \ne k' \in \{1,\ldots ,5\}$.

Challenge C4 requires us to estimate the probabilities $p_1=\text {Pr}\left( Y_{i}>s_i;\,i\in I\right)$ and $p_2=\text {Pr}(Y_{i}>s_1;\, i \in I)$, where $s_i := \mathbbm {1}(i \in \{1,2,\ldots ,25\})s_1 + \mathbbm {1}(i \in \{26,27,\ldots ,50\})s_2$ and $s_1$ ($s_2$) denotes the marginal level exceeded once every year (month) on average. Under the assumption of independence between groups, the challenge can be broken down to 5 lower-dimensional challenges involving the estimation of joint tail probabilities for each $G_k,$ $k \in \{1,\ldots ,5\}.$ These can then be multiplied together to obtain the required overall probabilities due to (assumed) between-group independence; specifically, we have $p_1= \prod _{k=1}^5\text {Pr}\left( Y_{i} >s_i ;\ i\in G_k\right)$ and $p_2=\prod _{k=1}^5\text {Pr}\left( Y_{i} >s_1 ;\ i\in G_k\right)$.

### Conditional extremes

The conditional multivariate extreme value model (CMEVM) of Heffernan and Tawn (2004) provides a flexible multivariate extreme value framework capable of capturing a range of extremal dependence forms without making assumptions about the specific form of joint dependence structure. Consider a *d*-dimensional random variable $\varvec{W}=(W_1, \ldots , W_d)$ on standard Laplace margins. For $i\in \{1,\ldots , d\}$, the CMEVM approach assumes the existence of parameter vectors $\varvec{\alpha }_{-|i} \in [-1, 1]^{d-1}$ and $\varvec{\beta }_{-|i} \in (-\infty , 1]^{d-1}$ such that

$$\begin{aligned} \lim _{u_i \rightarrow \infty }\operatorname {Pr}\left\{ \varvec{W}_{-i} \le \varvec{\alpha }_{-|i} W_i+W_i^{\varvec{\beta }_{-|i}} \varvec{z}_{\mid i}, W_i - u_i> w \mid W_i>u_i\right\} =e^{-w}H_{\mid i}\left( \varvec{z}_{\mid i}\right) , \quad w >0, \end{aligned}$$

with non-degenerate distribution function $H_{\mid i}(\cdot ),$ vector operations being applied componentwise, and conditional threshold $u_i$. The vector $\varvec{W}_{-i}$ denotes $\varvec{W}$ excluding its $i^{\text {th}}$ component and $\varvec{z}_{\mid i}$ is within the support of the residual random vector $\varvec{Z}_{\mid i}=(\varvec{W}_{-i}-\varvec{\alpha }_{-|i}w_i)/{w_i^{\varvec{\beta }_{-|i}}}\sim H_{\mid i}(\cdot )$. We apply this model to data where $W_i>u_i$, for some finite conditioning threshold $u_i$, to estimate the probabilities $p_1$ and $p_2$ defined in Section 5.1, using the inference procedure of Keef et al. (2013a).

### Results

Let $\varvec{W}:=(W_1, \ldots , W_{50})$ denote the random vector after transformation to standard Laplace margins. This vector is divided into the five subgroups identified in Section 5.1, and the subgroup probabilities are estimated using predictions obtained from the sampling method of Heffernan and Tawn (2004). We condition on the first variable of each subgroup being extreme, and simulate $10^8$ predictions from each of the resulting fitted conditional extremes models. To account for uncertainty in the estimates, we perform a parametric bootstrapping procedure with 100 samples.

Sensitivity analyses of the estimated probabilities to the choice of conditioning variable suggest no significant effect. Furthermore, we consider a range of conditioning thresholds; the corresponding estimates of subgroup probabilities defined in Section 5.1 appear relatively stable with respect to the conditioning threshold quantile. We ultimately select 0.85-quantiles for the conditioning thresholds of our final probability estimates. These are given by $\hat{p}_1 = 1.094\times 10^{-26}$ $(2.150\times 10^{-36}, 1.359\times 10^{-24})$ and $\hat{p}_2 = 1.076\times 10^{-31}$ $(1.596\times 10^{-46}, 1.850\times 10^{-29})$, with 95% confidence intervals obtained from parametric bootstrapping given in parentheses.

## Discussion

In this paper, we have proposed a range of statistical methods for estimating extreme quantities for challenges C1-C4. For the univariate challenge C1, we estimated the 0.9999-quantile, and the associated 50% confidence intervals, of $Y\mid \varvec{X} = \varvec{x_{i}}$, $i \in \{1,\ldots ,n\}$. For challenge C2, we estimated a quantile, corresponding to a once in 200 year level, of the marginal distribution *Y* whilst incorporating the loss function in Eq. 3.2. Overall we ranked $6^{\text {th}}$ and and $4^{\text {th}}$ for challenges C1 and C2, respectively.

For challenge C1, our final model (model 7 in Table 1) was chosen to minimise the model selection criteria; however, QQ plots showed over-estimation of the most extreme values of the response (see Fig. 2). As a result, the conditional quantiles calculated for C1 are generally over-estimated when compared with the true quantiles. If we ignored the model selection criteria and chose the model based on a visual assessment of QQ plots, we would have chosen model 5 in Table 1 and this would have covered the true quantile on fewer occasions than our chosen model. Therefore, the main issue with our results concerns the width of the confidence intervals.

Narrow confidence intervals are an indication of over-fitting and this could have arisen in several places. For instance, Rohrbeck et al. (2023) suggested all the seasonality is captured in the threshold, while our model includes a seasonal threshold and a covariate for seasonality in the scale parameter of the GPD model. As well as over-fitting, the model may not have been flexible enough; this could be, in part, due to our model missing covariates. For instance, the true model contained $V_{2}$ as a covariate (Rohrbeck et al. 2023) whilst our model did not. In addition, the basis dimensions for our splines are low. In practice, a higher dimension than we would expect should be considered and, although we chose the dimension using a model-based approach, it may have resulted in the splines not being flexible enough to capture all of the trends in the data.

Narrow confidence intervals may have also resulted from the choice of uncertainty quantification procedure. Changing the average block length *l* in our stationary bootstrap procedure would alter the confidence interval widths, although this was carefully chosen to reflect the temporal dependence in the data. Alternative methods, such as the standard bootstrap procedure or the delta method, could be implemented to investigate how this affects the confidence interval widths. We expect that such confidence intervals will be wider than those presented here since the dependence in the data is not accounted for, but assuming temporal independence would be inaccurate. Therefore, whilst adopting an alternative procedure may widen confidence intervals, thus improving our performance, such intervals may not be well calibrated for this data set.

The over-fitting and over-estimation issues encountered in C1 are carried through to C2 since the same model is used for both challenges. However, one aspect specific to C2 is the choice of quantile evaluation within the loss function. Many methods exist for evaluating the non-stationary quantiles which feed into the loss function term of the objective function $S(\varvec{\theta })$ in Eq. 3.5. As the loss function will be dominated by the log-likelihood in $S(\varvec{\theta })$, we choose to transform to standard exponential margins when evaluating the quantiles in order to give more importance to the loss function. Since the data is light tailed ($\xi < 0$) this transformation elongates the tail and therefore inflates any deviations between the model and theoretical quantiles which in turn, inflates the contribution of the average loss function to $S(\varvec{\theta })$. However, this approach means that the objective function will have a preference to minimise the deviations in the upper-tail of the distribution, leading to potential over-fitting to the upper-tail and possibly, a poor fit in the rest of the tail. This may not necessarily be undesirable since the loss function penalises under-estimation more than over-estimation, however, since the model in C1 already over-fits, this method may only exacerbate the problem for C2.

For the first multivariate challenge C3, we employed an extension of the method proposed by Wadsworth and Tawn (2013) to estimate probabilities of three variables lying in extremal sets. Our extension accounts for non-stationarity in the extremal dependence structure, with GAMs used to represent covariate relationships. The QQ plots for the resulting model suggested reasonable fits. For this challenge, we ranked 5^th^ and our estimates are on the same order of magnitude as the truth (Rohrbeck et al. 2023).

We note similarities in the methodologies presented for the challenges C1, C2, and C3. Specifically, each of the proposed methods used the EVGAM framework for capturing non-stationary tail behaviour via a generalised Pareto distribution. We acknowledge that the model selection tool proposed for C1 and C2 could also be applied for C3. However, we opted not to use this tool for several reasons. Firstly, unlike the univariate setting, there is no guarantee of convergence to a GPD in the limit, and the GPD tail assumption thereby needs to be tested. Moreover, in exploratory analysis, we tested the model selection tool for C3 but found the selected models and quantiles to not be satisfactory, particularly in the upper tail of the min-projection variable. We therefore selected a model manually, using QQ plots to evaluate performance. Exploring threshold and model selection techniques for multivariate extremes represents an important area of research.

In the final multivariate challenge C4, we estimated very high-dimensional joint survival probabilities. To do so, we split the probability into 5 lower-dimensional components which are assumed independent of each other, then estimated each using the CMEVM of Heffernan and Tawn (2004). In the final rankings of Rohrbeck et al. (2023), we ranked $3^{\text {rd}}$ for this challenge. A more prudent method could have been implemented, as groups of variables were never truly independent. Alternatively, although we achieve relatively stable probability estimates with respect to threshold in Section 5.2 (see Supplementary Material for details), our approach could potentially have been improved by estimating individual group probabilities across varying thresholds and taking an average value as our final result. We also do not report the effect of the choice of the conditioning variable on our estimates. Preliminary analysis suggested this to be negligible. However, conditioning on each site in a given subgroup and then taking a weighted sum of the resulting probabilities (e.g., Keef et al. 2013b) may have resulted in more robust estimates.

## Supplementary Information

**Supplementary Material for “Extreme value methods for estimating rare events in Utopia”**

## Supplementary Information

Below is the link to the electronic supplementary material.

Supplementary file 1 (pdf 10031 KB)

## Data Availability

The data for the EVA (2023) Conference Data Challenge has been made publicly available by Rohrbeck et al. (2024).

## Code Availability

Code supporting the findings of this study is available from the corresponding author upon reasonable request.

## Materials Availability

Materials that support the findings of this study are available from the corresponding author upon reasonable request.

## References

[CR1] Bernard, E., Naveau, P., Vrac, M., Mestre, O.: Clustering of maxima: spatial dependencies among heavy rainfall in France. J. Clim. **26**, 7929–7937 (2013)

[CR2] Chavez-Demoulin, V., Davison, A.C.: Generalized additive modelling of sample extremes. J. R. Stat. Soc. Ser. C Appl. Stat. **54**, 207–222 (2005)

[CR3] Coles, S.: An Introduction to Statistical Modeling of Extreme Values. Springer, London (2001)

[CR4] D’Arcy, E., Tawn, J.A., Joly, A., Sifnioti, D.E.: Accounting for seasonality in extreme sea-level estimation. Ann. Appl. Stat. **17**(4), 3500–3525 (2023)

[CR5] Davison, A.C., Smith, R.L.: Models for exceedances over high thresholds. J. R. Stat. Soc. Ser. B Stat. Methodol. **52**, 393–425 (1990)

[CR6] Eastoe, E.F., Tawn, J.A.: Modelling non-stationary extremes with application to surface level ozone. J. R. Stat. Soc. Ser. C Appl. Stat. **58**, 25–45 (2009)

[CR7] Gneiting, T., Katzfuss, M.: Probabilistic forecasting. Ann. Rev. Stat. App. **1**, 125–151 (2014)

[CR8] Guerrero, M.B., Huser, R., Ombao, H.: Conex–Connect: learning patterns in extremal brain connectivity from multichannel EEG data. Ann. Appl. Stat. **17**, 178–198 (2023)

[CR9] Hastie, T., Tibshirani, R., Friedman, J.: The elements of statistical learning. Springer, New York (2001)

[CR10] Heffernan, J.E., Tawn, J.A.: A conditional approach for multivariate extreme values. J. R. Stat. Soc. Ser. B Stat. Methodol. **66**, 497–546 (2004)

[CR11] Hill, B.M.: A simple general approach to inference about the tail of a distribution. Ann. Stat. **3**, 1163–1174 (1975)

[CR12] Joe, H.: Multivariate models and multivariate dependence concepts. Chapman and Hall/CRC, New York (1997)

[CR13] Jonathan, P., Randell, D., Wu, Y., Ewans, K.: Return level estimation from non-stationary spatial data exhibiting multidimensional covariate effects. Ocean Eng. **88**, 520–532 (2014)

[CR14] Keef, C., Papastathopoulos, I., Tawn, J.A.: Estimation of the conditional distribution of a multivariate variable given that one of its components is large: additional constraints for the Heffernan and Tawn model. J. Multivar. Anal. **115**, 396–404 (2013)

[CR15] Keef, C., Tawn, J.A., Lamb, R.: Estimating the probability of widespread flood events. Environmetrics **24**, 13–21 (2013)

[CR16] Kyselý, J., Picek, J., Beranová, R.: Estimating extremes in climate change simulations using the peaks-over-threshold method with a non-stationary threshold. Glob. Planet. Chang. **72**, 55–68 (2010)

[CR17] Ledford, A.W., Tawn, J.A.: Statistics for near independence in multivariate extreme values. Biometrika **83**, 169–187 (1996)

[CR18] Mhalla, L., Opitz, T., Chavez-Demoulin, V.: Exceedance-based nonlinear regression of tail dependence. Extremes **22**, 523–552 (2019)

[CR19] Murphy, C., Tawn, J.A., Varty, Z.: Automated threshold selection and associated inference uncertainty for univariate extremes. arXiv:2310.17999 (2024)

[CR20] Murphy-Barltrop, C., Wadsworth, J.: Modelling non-stationarity in asymptotically independent extremes. Comput. Stat. Data Anal. **199**, 108025 (2024)

[CR21] Northrop, P.J., Jonathan, P.: Threshold modelling of spatially dependent non-stationary extremes with application to hurricane-induced wave heights. Environmetrics **22**, 799–809 (2011)

[CR22] Pickands, J.: Statistical inference using extreme order statistics. Ann. Stat **3**, 119–131 (1975)

[CR23] Politis, D.N., Romano, J.P.: The stationary bootstrap. J. Am. Stat. Assoc. **89**, 1303–1313 (1994)

[CR24] Resnick, S.: Hidden regular variation, second order regular variation and asymptotic independence. Extremes **5**, 303–336 (2002)

[CR25] Rohrbeck, C., Simpson, E.S., Tawn, J.A.: Editorial: EVA (2023) Conference Data Challenge. Extremes, (to appear). (2023)

[CR26] Rohrbeck, C., Simpson, E.S., Tawn, J.A.: Dataset for EVA 2023 data challenge. Bath: University of Bath Research Data Archive, (in press). (2024)

[CR27] Simpson, E.S., Wadsworth, J.L., Tawn, J.A.: Determining the dependence structure of multivariate extremes. Biometrika **107**, 513–532 (2020)

[CR28] Wadsworth, J.L., Tawn, J.A.: A new representation for multivariate tail probabilities. Bernoulli **19**, 2689–2714 (2013)

[CR29] Wood, S.N.: Generalized additive models. Chapman and Hall/CRC, New York (2017)

[CR30] Youngman, B.D.: Generalized additive models for exceedances of high thresholds with an application to return level estimation for U.S. wind gusts. Journal of the American Statistical Association, (2019) **114**, 1865–1879 (2019)

[CR31] Youngman, B.D.: evgam: An R package for generalized additive extreme value models. J. Stat. Softw. **103**(3), 1–26 (2022)

[CR32] Yu, K., Moyeed, R.A.: Bayesian quantile regression. Stat. Probab. Lett. **54**, 437–447 (2001)

