# Supplementary material for: Extreme value methods for estimating rare events in Utopia: EVA (2023) conference data challenge: team Lancopula Utopiversity
Source: Extremes (Boston). 2024 Nov 22;28(1):23–45. doi: 10.1007/s10687-024-00498-w (PMC11996970; doi:10.1007/s10687-024-00498-w)
Supplement: Supplementary file 1 — (pdf 10031 KB) [file 10687_2024_498_MOESM1_ESM.pdf]

Supplementary Material for “Extreme value methods for estimating rare events in Utopia”

Lídia Maria André[1], Ryan Campbell[2], Eleanor D’Arcy[3], Aiden Farrell[2], Dáire Healy[4], Lydia Kakampakou[2,*], Conor Murphy[1], Callum John Rowlandson Murphy-Barltrop[5,6] and Matthew Speers[1]

[1]STOR-i Centre for Doctoral Training, Lancaster University LA1 4YR, United Kingdom
[2]School of Mathematical Sciences, Lancaster University LA1 4YF, United Kingdom
[3]Environment Agency, Lutra House, Dodd Way Off Seedlee Road, Walton Summit Centre, Preston PR5 8BX, United Kingdom
[4]Dipartimento di Scienze Ambientali, Informatica e Statistica, Università Ca’ Foscari Venezia, Campus Scientifico, via Torino 155, Mestre 30172, Italia
[5]Technische Universität Dresden, Institut Für Mathematische Stochastik, Helmholtzstraße 10, 01069 Dresden, Germany.
[6]Center for Scalable Data Analytics and Artificial Intelligence (ScaDS.AI) Dresden/Leipzig, Germany
[*]Correspondence to: l.kakampakou1@lancaster.ac.uk

September 2, 2024

S.1 Additional figures for Section 3

In this section, we present additional figures for Section 3 of the main paper, concerned with challenges C1 and C2. Figures S1-S3 support the exploratory analysis for challenges C1 and C2. We explore the within-year seasonality of the response variable Y in Figure S1, looking at the distribution of Y per month and across the two seasons. This shows that there is a significant difference in the distribution of Y between seasons 1 and 2, but within each season there is little difference across months.

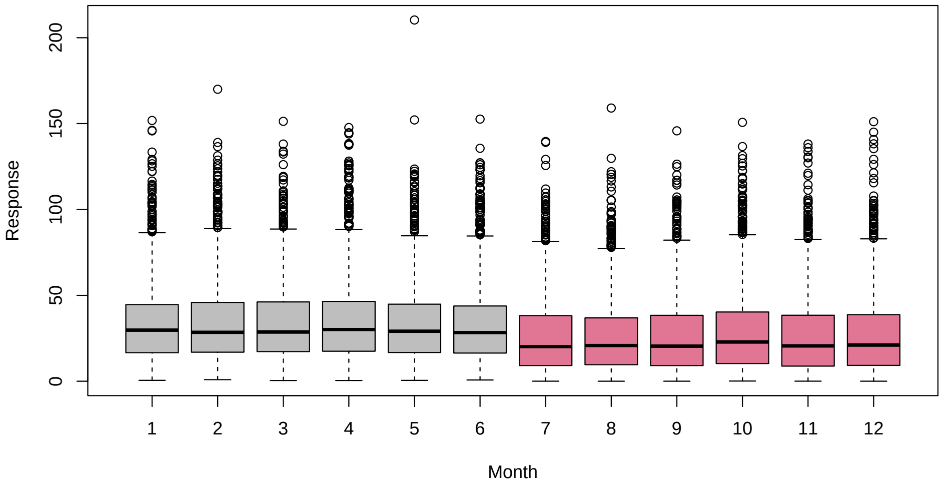

Figure S1: Box plot of the response variable Y with each month and season (season 1 in grey and season 2 in red).

Figure S2 shows a scatter plot of Y against each covariate $V_1,\dots,V_8$, excluding V_6 which corresponds to season. Covariates V_1, V_2 and V_8 do not seem to have a relationship with Y, whilst there seems to be dependence for the remaining covariates. These observed relationships appear complex and non-linear.

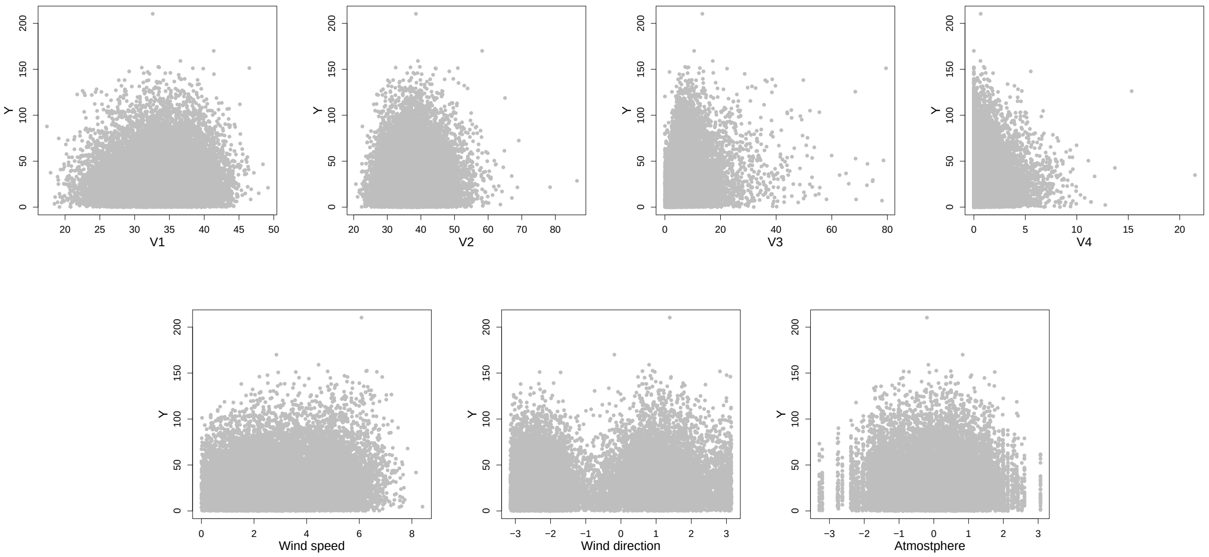

Figure S2: Scatter plots of explanatory variables $V_1,\ldots,V_4$, wind speed (V_6), wind direction (V_7) and atmosphere (V_8), from top-left to bottom-right (by row), against the response variable Y.

We also explore temporal dependence in Figure S3 that details the auto-correlation function (acf) values for the response Y and explanatory variables $V_1,\ldots,V_4,V_6,\ldots,V_8$, up to a lag of 60. All variables have negligible acf values beyond lag 0, except V_6 (wind speed), V_7 (wind direction) and V_8 (atmosphere).

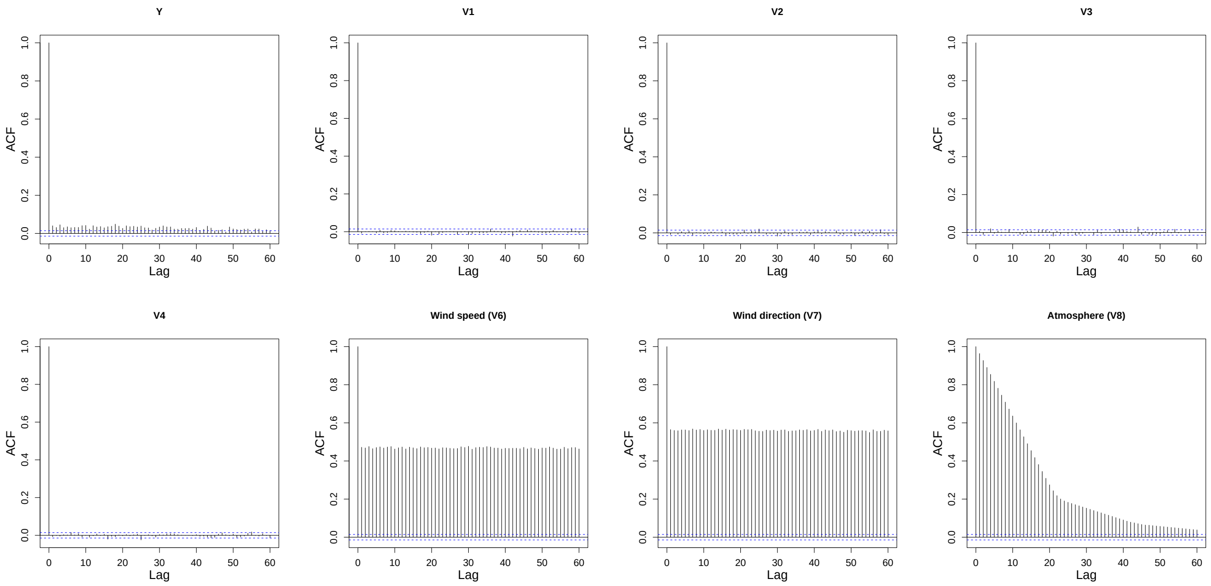

Figure S3: Autocorrelation function plots for the response variable Y and explanatory variables $V1,\ldots,V4$, wind speed ($V6$), wind direction ($V7$) and atmosphere ($V8$), from top-left to bottom-right (by row).

Figure S4 shows the QQ-plots corresponding to a standard GPD model fitted to the excesses of Y above a constant (left) and seasonally-varying threshold (right). 95% tolerance bounds (grey) show a lack of agreement between observations and the standard GPD model above a constant threshold. The second plot demonstrates a significant improvement in model fit.

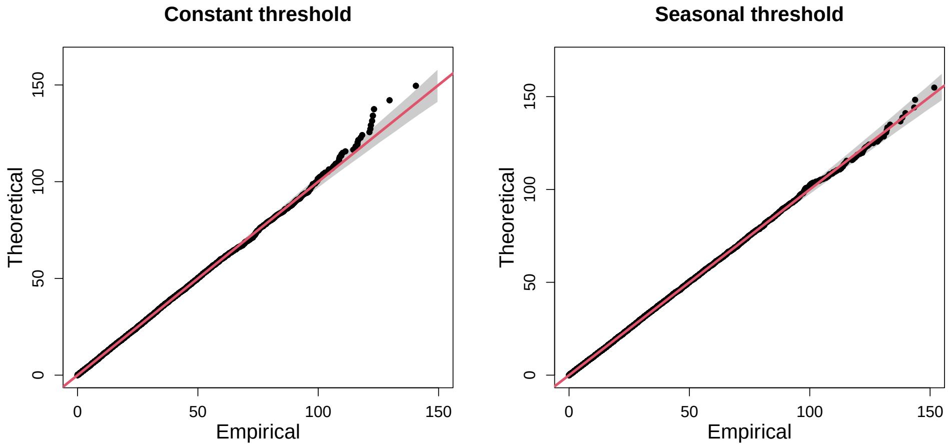

Figure S4: QQ-plots showing standard GPD model fits with 95% tolerance bounds (grey) above a constant (left) and stepped-seasonal (right) threshold.

Figure S5 shows a detailed summary of the pattern of missing data in the data and can be

produced using the `missing_pattern` function in the `finalfit` package in `R` (Harrison et al., 2023). To interpret the figure note that blue and red squares represent observed and missing variables, respectively. The number on the right indicates the number of missing predictor variables (i.e., the number of red squares in the row), while the number on the left is the number of observations that fall into the row category. On the bottom, we have the number of observations that fall into the column category. For example, 18,545 observations are fully observed (denoted by the first row); there are 407 observations where only $V4$ is missing (denoted by the second row), 13 observations where both $V4$ and $V6$ are missing (denoted by the fourth row), 456 observations where $V4$ and at least one other predictor is missing (denoted by the last column in the table), etc. It can be seen that there are very few observations where more than one predictor is missing.

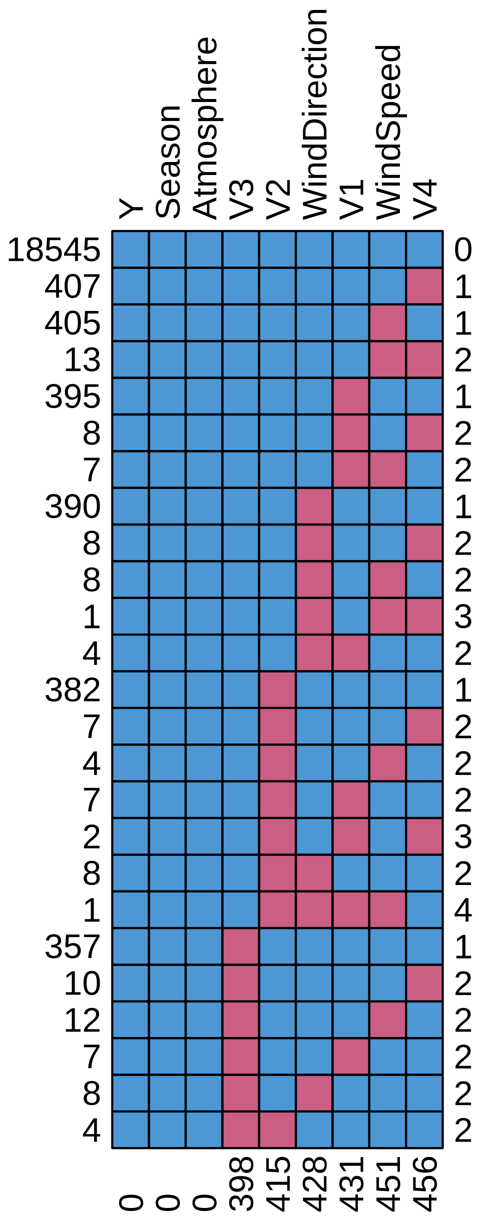

Figure S5: Detailed pattern of missing predictor variables in the Amaurot data set.

S.2 Additional figures for Section 4

In this section, we present additional plots related to Section 4 of the main article. Figure S6 illustrates the time series of both covariates for the first 3 years of the observation period. It can be seen how the seasons vary periodically over each year, as well as the discrete nature of the atmospheric covariate.

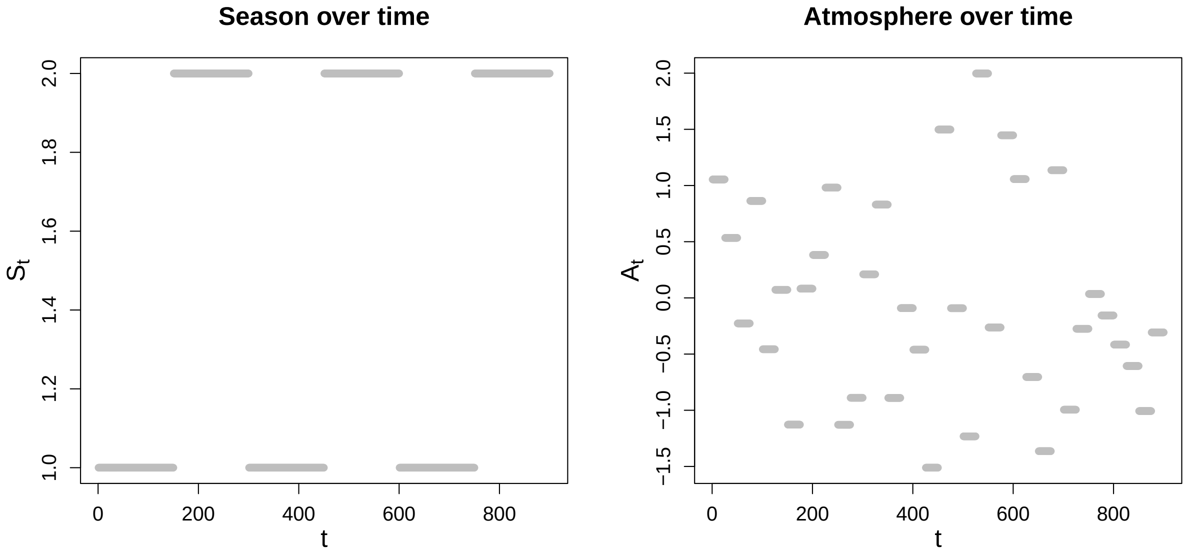

Figure S6: Plots of S_t (left) and A_t (right) against t for the first 3 years of the observation period.

Bootstrapped χ estimates for the groups $G^A_{I,k}, k \in \{1,\ldots,10\}, I \in \mathscr{I} \setminus \{1,2,3\}$ and $G^S_{I,k}, k \in \{1,2\}, I \in \mathscr{I}$ are given in Figures S7 - S10. These estimates illustrate the impact of atmosphere on the dependence structure.

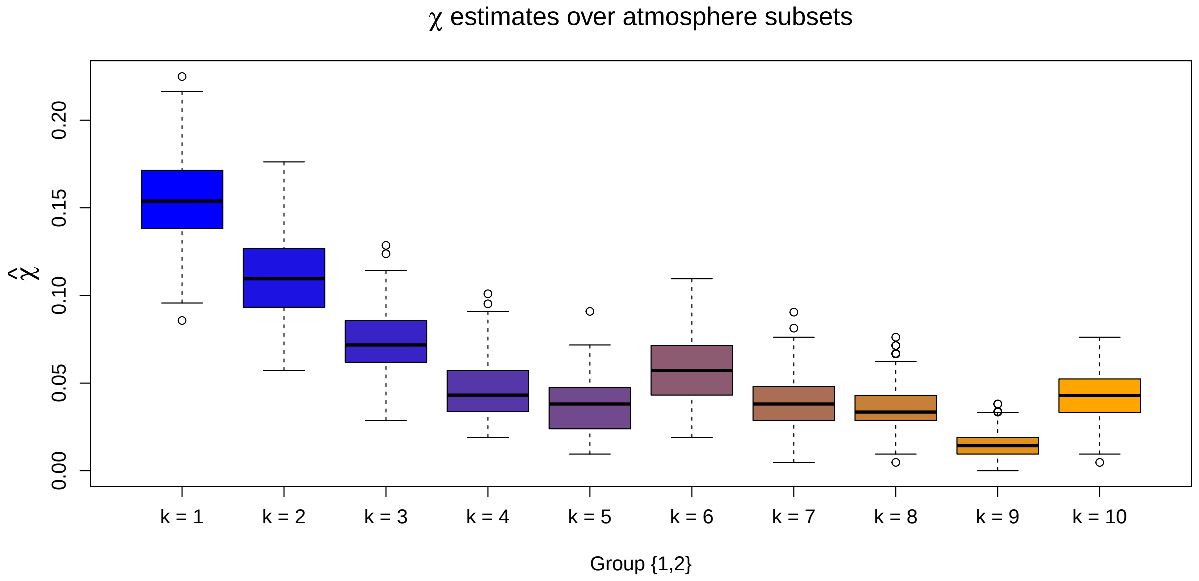

Figure S7: Boxplots of empirical χ estimates obtained for the subsets $G^A_{I,k}$, with $k = 1,\ldots,10$ and $I = \{1,2\}$. The colour transition (from blue to orange) over k illustrates the trend in χ estimates as the atmospheric values are increased.

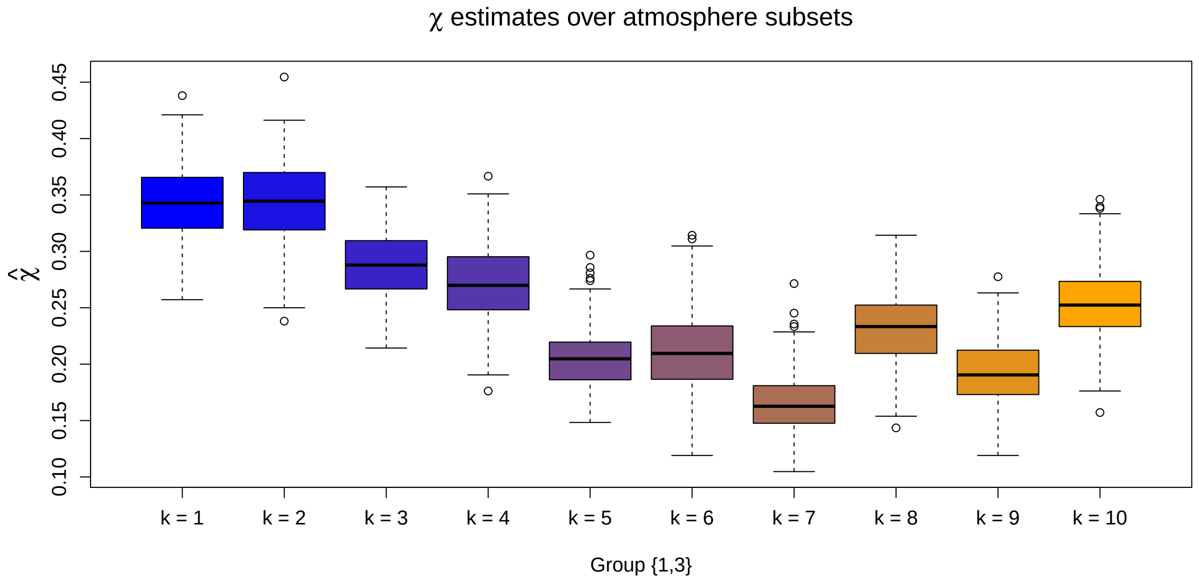

Figure S8: Boxplots of empirical χ estimates obtained for the subsets $G^A_{I,k}$, with $k = 1,\dots,10$ and $I = \{1,3\}$. The colour transition (from blue to orange) over k illustrates the trend in χ estimates as the atmospheric values are increased.

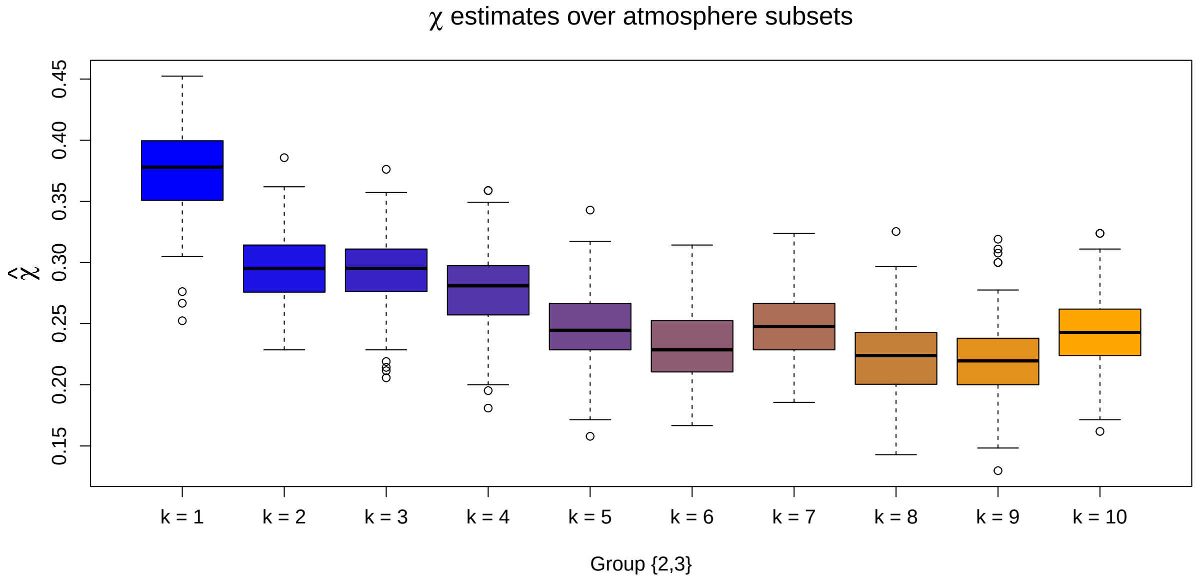

Figure S9: Boxplots of empirical χ estimates obtained for the subsets $G^A_{I,k}$, with $k = 1,\dots,10$ and $I = \{2,3\}$. The colour transition (from blue to orange) over k illustrates the trend in χ estimates as the atmospheric values are increased.

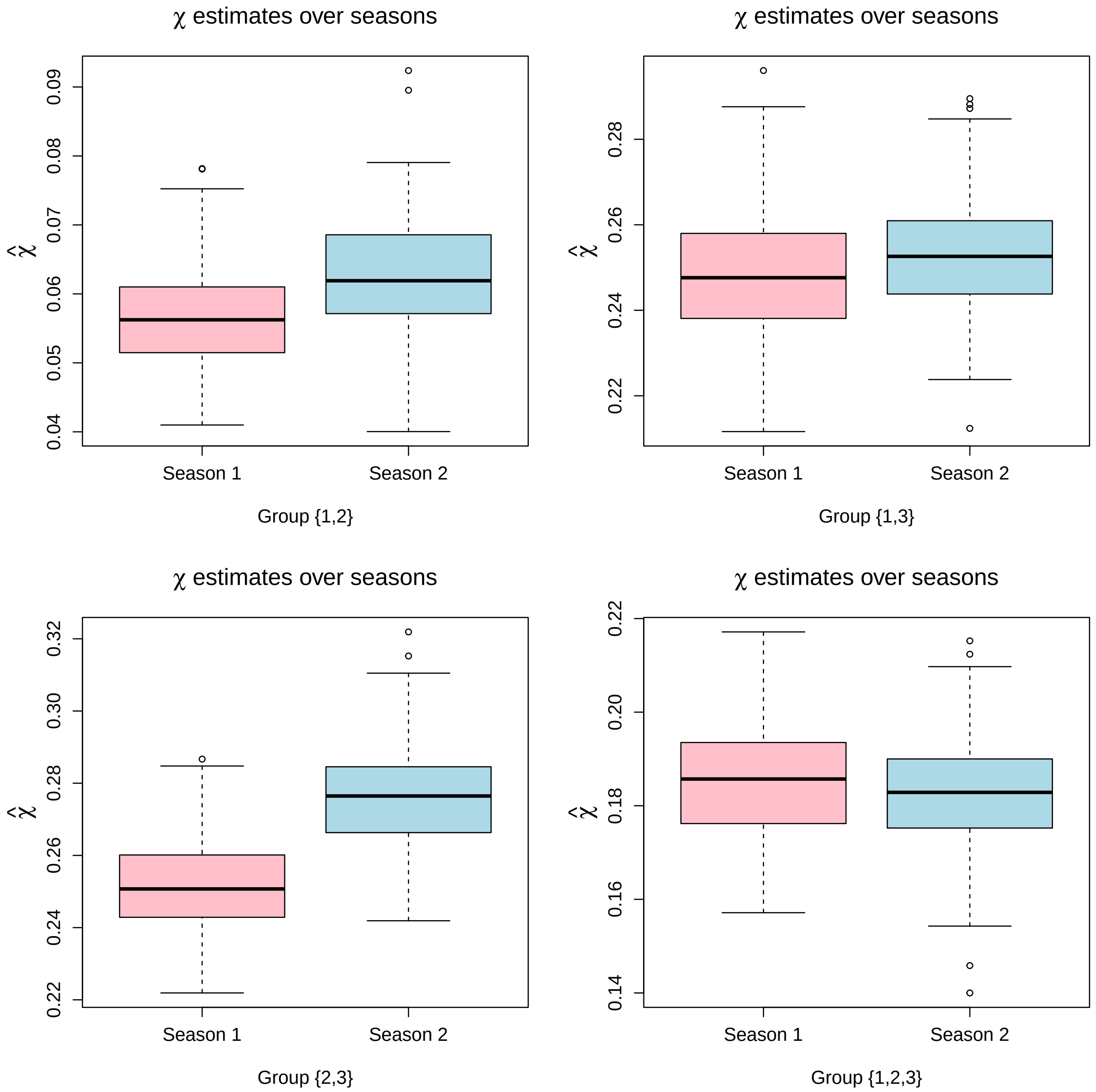

Figure S10: Boxplots of empirical χ estimates obtained for the subsets $G_{I,k}^{S}$, with $k = 1,2$. In each case, pink and blue colours illustrate estimates for seasons 1 and 2, respectively. From top left to bottom right: $I = \{1,2,3\}$, $I = \{1,2\}$, $I = \{1,3\}$, $I = \{2,3\}$.

For a 3-dimensional random vector, the angular dependence function, denoted $\lambda(\cdot)$, is defined on the unit-simplex $\mathcal{S}^2$ and describes extremal dependence along different rays $\boldsymbol{\omega} \in \mathcal{S}^2$. As noted in Section 4.2 of the main manuscript, we can associate each of the probabilities from C3, p_1 and p_2, with points on $\mathcal{S}^2$, denoted $\boldsymbol{\omega}^1$ and $\boldsymbol{\omega}^2$ respectively. With $I = \{1,2,3\}$, we consider $\lambda(\boldsymbol{\omega}^1)$ and $\lambda(\boldsymbol{\omega}^2)$ over the subsets $G^S_{I,k}$, $k \in \{1,2\}$ and $G^A_{I,k}$, $k \in \{1,\dots,10\}$. We note that $\lambda(\boldsymbol{\omega}^1)$ is analogous with the coefficient of tail dependence $\eta \in (0,1]$ (Ledford and Tawn, 1996), with $\eta = 1/3\lambda(\boldsymbol{\omega}^1)$; this corresponds with the region where all variables are simultaneously extreme. Furthermore, $\lambda(\boldsymbol{\omega}^2)$, which corresponds to a region where only two variables are extreme, is only evaluated after an additional marginal transformation of the third Coputopia time series; see Section 4.2 of the main manuscript.

Estimation of $\lambda(\cdot)$ for each simplex point and subset was achieved using the Hill estimator (Hill, 1975) at the 90% level, with uncertainty subsequently quantified via bootstrapping. These results are given in Figures S11 - S14. These plots provide further evidence of a relationship between the extremal dependence structure and the covariates.

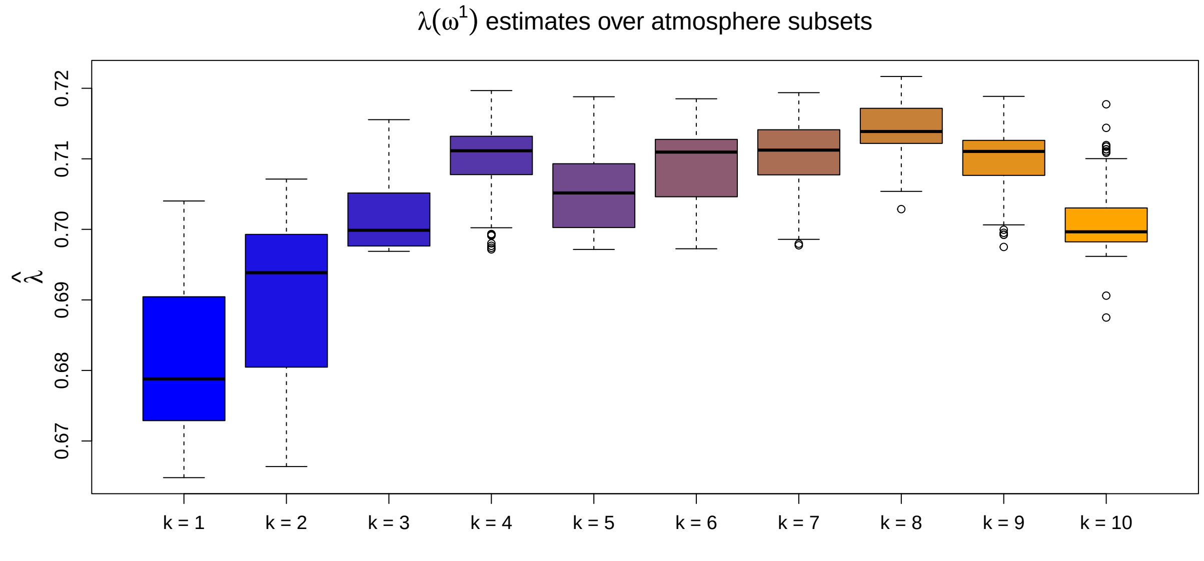

Figure S11: Boxplots of empirical $\lambda(\boldsymbol{\omega}^1)$ estimates obtained for the subsets $G^A_{I,k}$, with $k = 1,\dots,10$ and $I = \{1,2,3\}$. The colour transition (from blue to orange) over k illustrates the trend in λ estimates as the atmospheric values are increased.

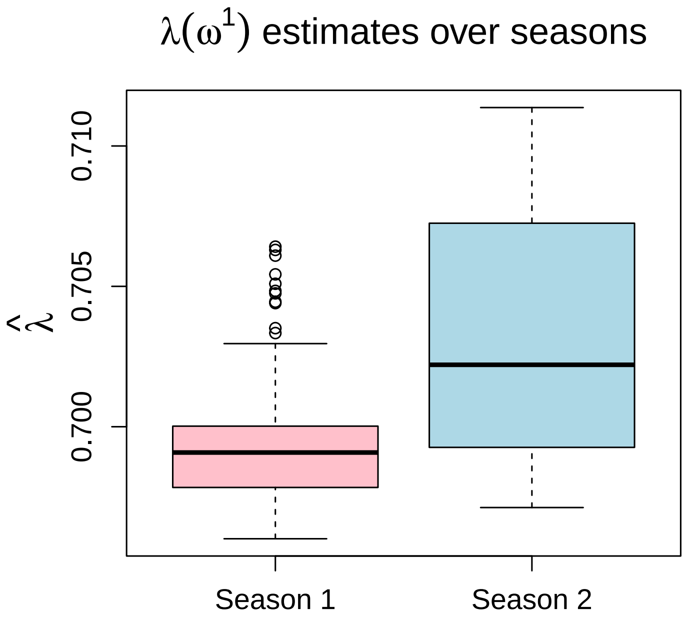

Figure S12: Boxplots of empirical $\lambda(\omega^1)$ estimates obtained for the subsets $G^S_{I,k}$, with $k = 1,2$ and $I = \{1,2,3\}$. In each case, pink and blue colours illustrate estimates for seasons 1 and 2, respectively.

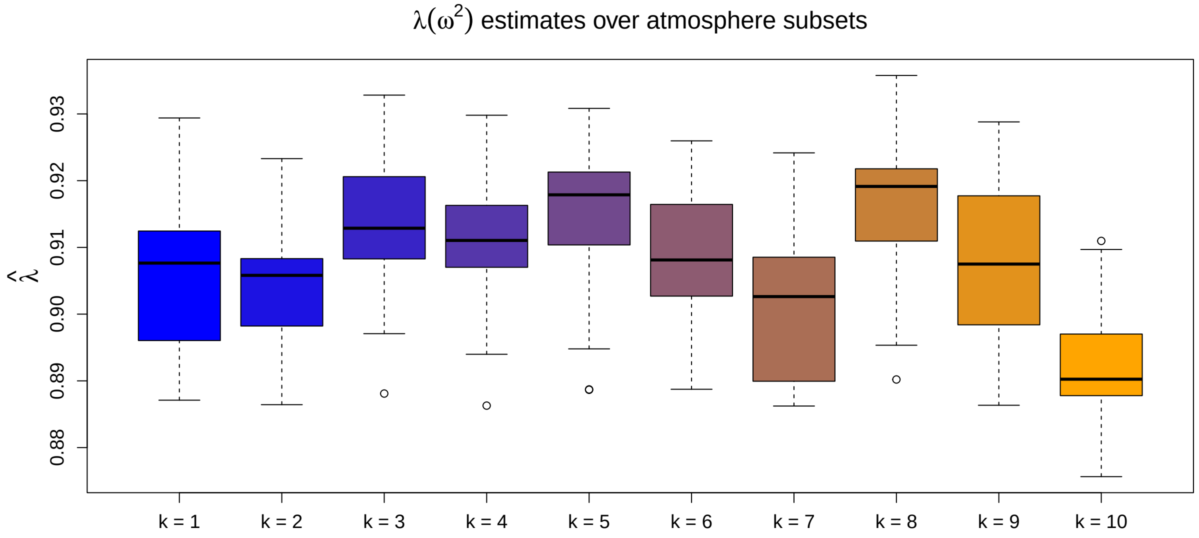

Figure S13: Boxplots of empirical $\lambda(\omega^2)$ estimates obtained for the subsets $G^A_{I,k}$, with $k = 1,\ldots,10$ and $I = \{1,2,3\}$. The colour transition (from blue to orange) over k illustrates the trend in λ estimates as the atmospheric values are increased.

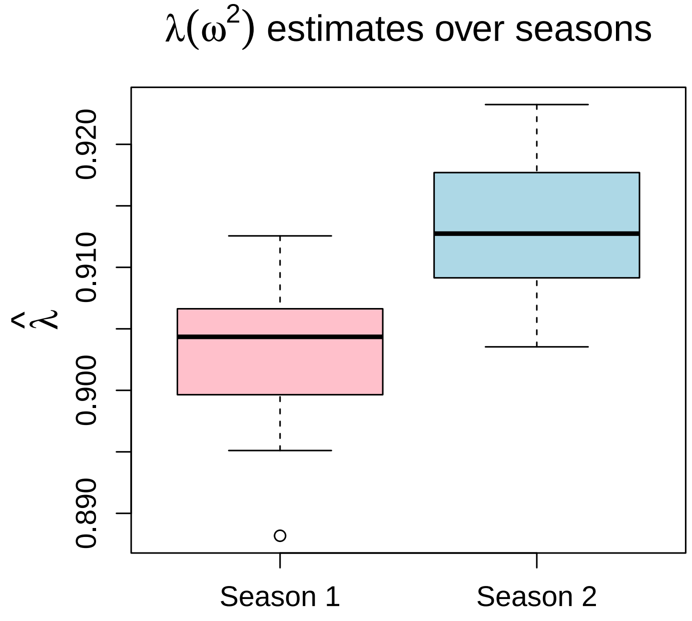

Figure S14: Boxplots of empirical $\lambda(\omega^2)$ estimates obtained for the subsets $G^S_{I,k}$, with $k = 1,2$ and $I = \{1,2,3\}$. In each case, pink and blue colours illustrate estimates for seasons 1 and 2, respectively.

To illustrate the estimated trend in dependence, Figure S15 shows the estimated scale functions, $\sigma(\boldsymbol{\omega};\boldsymbol{x}_t)$, over atmosphere for parts 1 and 2. Under the assumption of asymptotic normality in the spline coefficients, 95% confidence intervals are obtained via posterior sampling; see Wood (2017) for more details. We observe that σ tends to increase and decrease over atmosphere for parts 1 and 2, respectively, although the trend is less pronounced for the latter. Under our modelling framework, we note that higher values of σ are associated with less positive extremal dependence in the direction $\boldsymbol{\omega}$ of interest; to see this, observe that the survivor function of the GPD with fixed ξ is negatively associated with σ. Considering the trend in $\sigma(\boldsymbol{\omega};\boldsymbol{x}_t)$, our results indicate a decrease in dependence in the region where all variables are extreme.

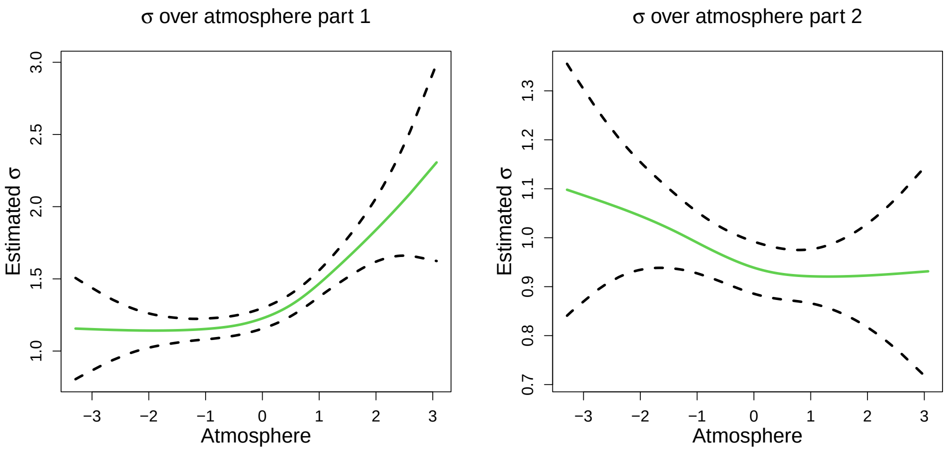

Figure S15: Estimated σ functions (green) over atmosphere for part 1 (left) and 2 (right). In both cases, the regions defined by the black dotted lines represent 95% confidence intervals obtained using posterior sampling.

S.3 Additional figures for Section 5

In this section, we present additional plots related to Section 5 of the main article an we refer to p_1 and p_2 as parts 1 and 2 of C4, respectively. Figure S16 shows a heat map of empirically estimated $\eta(\cdot)$ dependence coefficients and provides further evidence of the existence of the 5 dependence subgroups identified in our exploratory analysis for challenge C4. It also suggests that our modelling assumptions are reasonable; specifically that there is in-between group independence, and that the extremes within each group do not occur simultaneously.

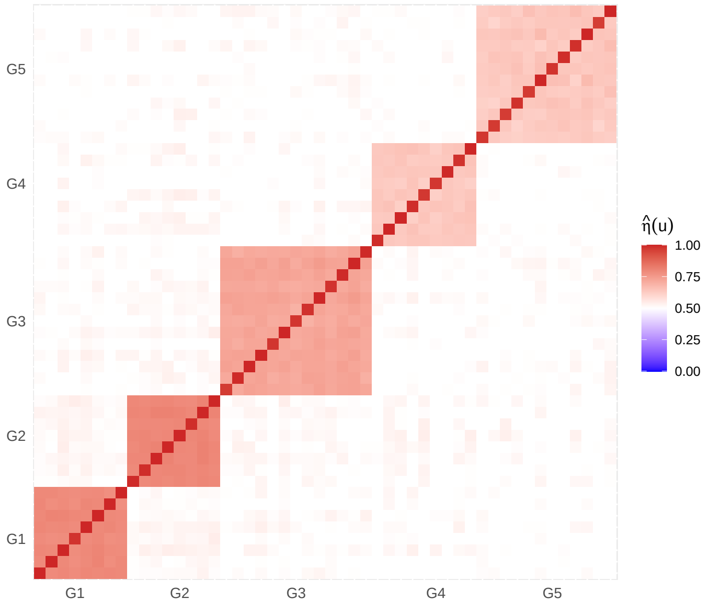

Figure S16: Heat map of estimated empirical pairwise $\eta(u)$ extremal dependence coefficients with $u = 0.95$.

Figure S17 shows the bootstrapped estimated individual group and overall probabilities with respect to conditioning threshold quantile for part 1 of challenge C4. Similarly, Figure S18 shows the bootstrapped estimated individual group and overall probabilities with respect to conditioning threshold quantile for part 2 of challenge C4.

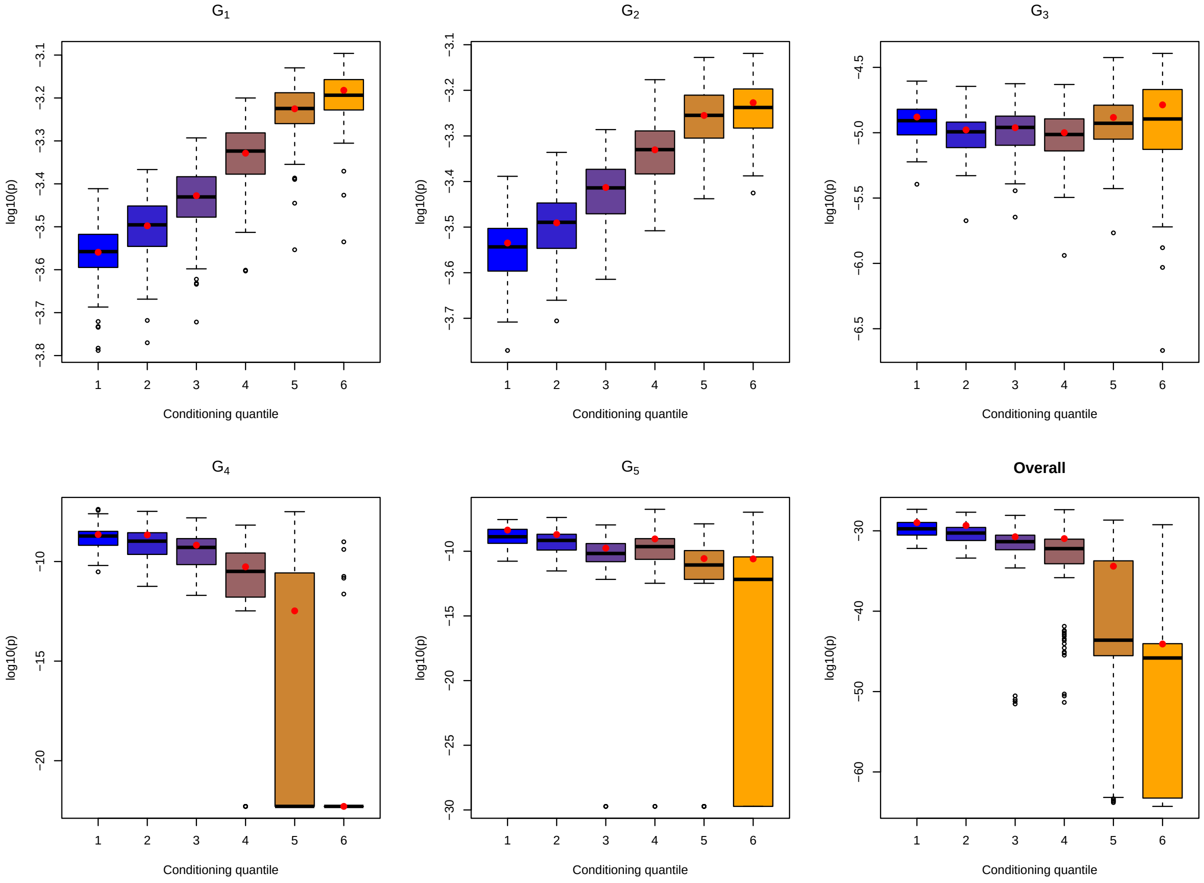

Figure S17: Part 1 subgroup and overall bootstrapped probability estimates on the log scale. The red points indicate the original sample estimates and the colouring of the boxplots indicates the choice of conditioning threshold, with the conditioning quantile indices 1-6 referring to the quantile levels $\{0.7, 0.75, 0.8, 0.85, 0.9, 0.95\}$, respectively.

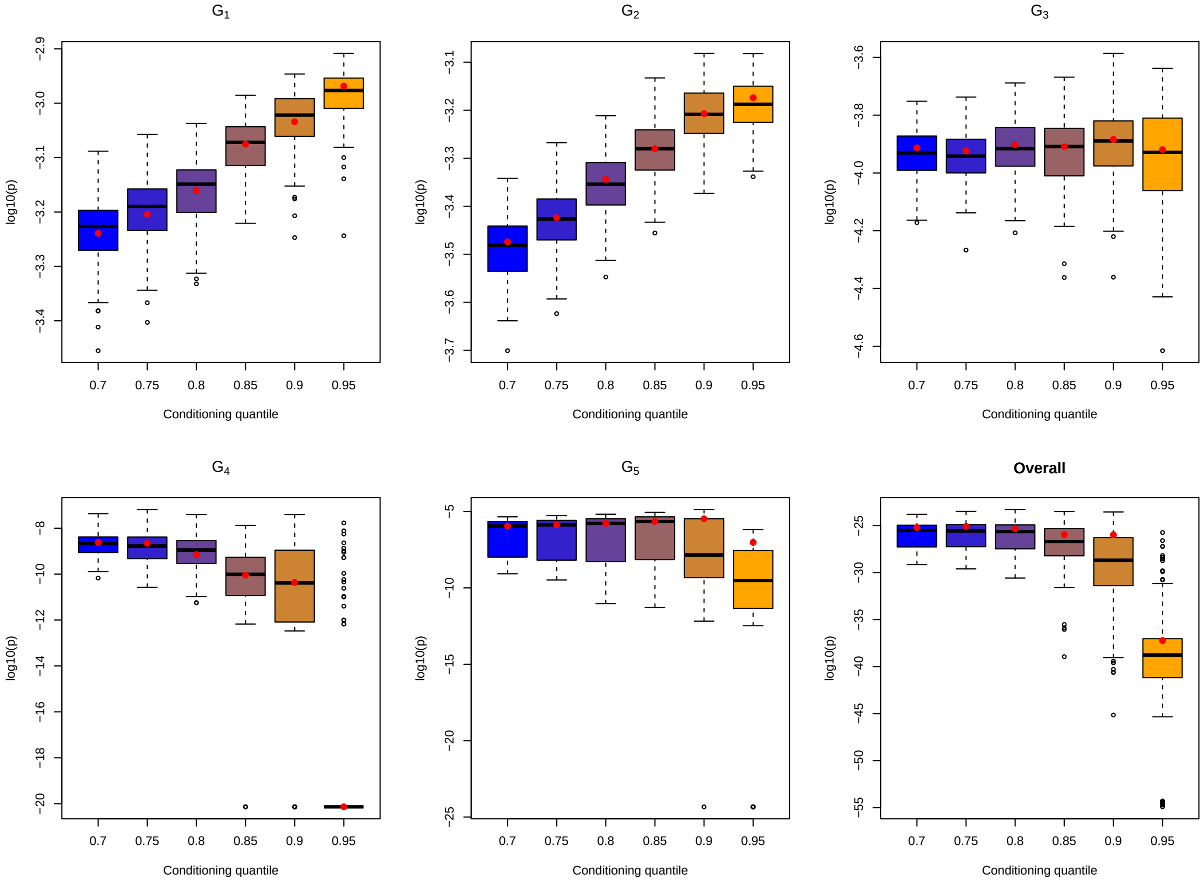

Figure S18: Part 2 subgroup and overall bootstrapped probability estimates on the log scale for C4. The red points indicate the original sample estimates and the colouring of the boxplots indicates the choice of conditioning threshold, with the conditioning quantile indices 1-6 referring to the quantile levels $\{0.7, 0.75, 0.8, 0.85, 0.9, 0.95\}$, respectively.

References

Harrison, E., Drake, T., and Ots, R. (2023). *finalfit: Quickly Create Elegant Regression Results Tables and Plots when Modelling*. R package version 1.0.7.

Hill, B. M. (1975). A simple general approach to inference about the tail of a distribution. *The Annals of Statistics*, 3:1163–1174.

Ledford, A. W. and Tawn, J. A. (1996). Statistics for near independence in multivariate extreme values. *Biometrika*, 83:169–187.

Wood, S. N. (2017). *Generalized additive models*. Chapman and Hall/CRC, New York.
